# Hydrogel‐Transformable Probiotic Powder for Targeted Eradication of *Helicobacter pylori* with Enhanced Gastric Mucosal Repair and Microbiota Preservation

**DOI:** 10.1002/advs.202500478

**Published:** 2025-03-16

**Authors:** Yongkang Lai, Hanchun Shen, Shige Wang, Yongliang Ouyang, Xinyuan Zhang, Bin Hu, Xiaoyi Zhang, Guisheng Li, Lizhi Xu, Jiulong Zhao

**Affiliations:** ^1^ Department of Gastroenterology, Shanghai Institute of Pancreatic Diseases, Changhai Hospital; National Key Laboratory of Immunity and Inflammation Naval Medical University Shanghai 200433 P. R. China; ^2^ School of Materials and Chemistry University of Shanghai for Science and Technology Shanghai 200093 P. R. China; ^3^ Department of Mechanical Engineering The University of Hong Kong Hong Kong SAR 999077 P. R. China; ^4^ Advanced Biomedical Instrumentation Centre Hong Kong Science Park Shatin, New Territories Hong Kong SAR 999077 P. R. China; ^5^ Materials Innovation Institute for Life Sciences and Energy (MILES) The University of Hong Kong Shenzhen Institute of Research and Innovation (HKU‐SIRI) Shenzhen 518057 P. R. China

**Keywords:** H. pylori, hydrogel, L. reuteri, probiotic powder, ROS‐responsive

## Abstract

*Lactobacillus reuteri* (*L. reuteri*) therapies represent a potentially effective approach to eradicating *Helicobacter pylori (H. pylori)*. However, the difficulty in bacterial viability preservation and harsh gastric environment compromises the survival and on‐target delivery of *L. reuteri*. This study presents a novel bacterium‐mediated bacterial elimination strategy using an edible *L. reuteri*@HTP probiotic powder for targeted bacterial elimination. The probiotic powder is obtained by grinding a lyophilized hydrogel composed of *L. reuteri*, hyaluronic acid (HA), tannic acid (TA), and polyvinyl alcohol (PVA). Upon contact with water, the powder quickly transforms into a hydrogel, enhancing *L. reuteri*’s survival in the harsh gastric environment and ensuring selective release at *H. pylori*‐infected inflammatory sites. *L. reuteri* targets and reduces *H. pylori* colonization while secreting reuterin to eliminate the bacteria. Additionally, TA's antioxidant properties help alleviate inflammation, and HA supports gastric mucosal repair. *L. reuteri*@HTP powder preserves the integrity of the gut microbiota, facilitating the restoration of a healthy microbiome. In particular, the probiotic powder remains stable at room temperature for at least six months, providing a promising alternative to traditional antibiotics for *H. pylori* treatment. This strategy combines targeted eradication, mucosal healing, and microbiome restoration, offering a new approach to treating gastric infections.

## Graphical abstract

A multifunctional, edible, and hydrogel‐transformable probiotic powder is developed based on the “bacteria‐mediated bacterial eradication” strategy. By leveraging the targeted binding capability of *L. reuteri* to *H. pylori*, this approach achieves an integrated therapeutic effect, including bactericidality, anti‐inflammation, gastric mucosal repair, and gut microbiota protection. This strategy offers an antibiotic‐free approach for *H. pylori* eradication, contributing to gastric cancer prevention.

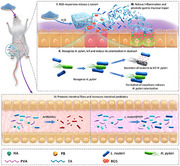

## 1 Introduction

*Helicobacter pylori* (*H. pylori*) is a Gram–negative spiral bacterium that resides in the stomach and affects nearly half of the world's population.^[^ ^1^ ^]^ *H. pylori* infection can lead to gastrointestinal diseases, including chronic gastritis, peptic ulcer disease, and gastric malignancies.^[^ ^2^ ^]^ Our previous large‐scale multicenter studies have provided substantial evidence linking *H. pylori* to gastric cancer.^[^ ^3^ ^]^ Additionally, a consensus in Kyoto in 2015 recommended that all *H. pylori*‐infected individuals undergo eradication therapy unless contraindications exist.^[^ ^4^ ^]^ Therefore, treating chronic *H. pylori* infection effectively has become essential for safeguarding global public health. Currently, regimens for *H. pylori* eradication are mainly based on a quadruple therapy regimen, which includes two kinds of antibiotics (i.e., amoxicillin and clarithromycin). However, limitations like antibiotic resistance and gut microbiota homeostasis disruption are associated with traditional antibiotic‐*H. pylori* eradication is becoming noticeably evident.^[^ ^5^ ^]^ Consequently, the management of *H. pylori* infection has become increasingly challenging, highlighting an urgent need for novel *H. pylori* infection eradication strategies.

Bacterium‐mediated bacterial elimination, characterized by precise targeting and intrinsic immunomodulatory effects, has garnered significant attention in biomedical research and innovation.^[^ ^6^ ^]^ *Lactobacillus reuteri* (*L. reuteri*) is a Gram–positive probiotic that is universally present in the intestinal tract of mammals. *L. reuteri* has been designated as a “Generally Recognized as Safe” food ingredient by the U.S. Food and Drug Administration and is widely used as a dietary supplement, demonstrating a high level of safety.^[^ ^7^ ^]^ *L. reuteri* can secrete a broad‐spectrum antimicrobial substance known as reuterin, showing potential as an innovative antibiotic agent.^[^ ^8^ ^]^ Particularly, previous research has indicated that *L. reuteri* can specifically recognize and bind to the surface proteins of *H. pylori*, forming complexes that promote its clearance through gastrointestinal motility. This interaction reduces the *H. pylori* load in the stomach and may support an increase in beneficial gut microbiota.^[^ ^9^ ^]^ However, *L. reuteri* showed unsatisfactory *H. pylori* eradication results. This inadequacy may stem from the harsh gastric environment that compromises the viability of *L. reuteri*.^[^ ^10^ ^]^ Moreover, the preservation of *L. reuteri* viability and the effective on‐target delivery of *L. reuteri* remain significant challenges in current bacterial‐based therapies.^[^ ^11^ ^]^ Accordingly, developing successful *H. pylori* eradication strategies that preserve the biological activity of *L. reuteri* is crucial to effectively addressing these challenges.

Hydrogels are 3D cross‐linked networks composed of hydrophilic polymer chains.^[^ ^12^ ^]^ The hydrophilic nature of hydrogels helps maintain bacterial viability by providing a suitable environment to survive and function effectively and shielding active compounds from degradation in harsh environments.^[^ ^13^ ^]^ In addition, hydrogels have adjustable modulus and adhesive properties. Modulating these features can improve targeting, allowing effective retention at inflammatory sites without rapid clearance by gastric fluids, which is crucial for treating infections like *H. pylori*.^[^ ^14^ ^]^ However, their physical consistency makes them difficult to swallow, particularly for patients with dysphagia or other swallowing disorders.^[^ ^15^ ^]^ Moreover, due to environmental factors and changes in the hydrogel's physical properties, hydrogels often suffer from challenges in prolonged storage.^[^ ^16^ ^]^ In comparison, a probiotic powder formulation can notably enhance the bacteria's stability, extend shelf life, and facilitate easier consumption.^[^ ^17^ ^]^ Nevertheless, the powder formulation faces significant challenges in ensuring effectively controlled and on‐target bacteria within the gastric milieu.^[^ ^18^ ^]^ Therefore, developing a hydrogel‐transformable powder containing *L. reuteri* could integrate the benefits of both hydrogel and powder and show promise for effectively eradicating *H. pylori*.

**Scheme 1:**
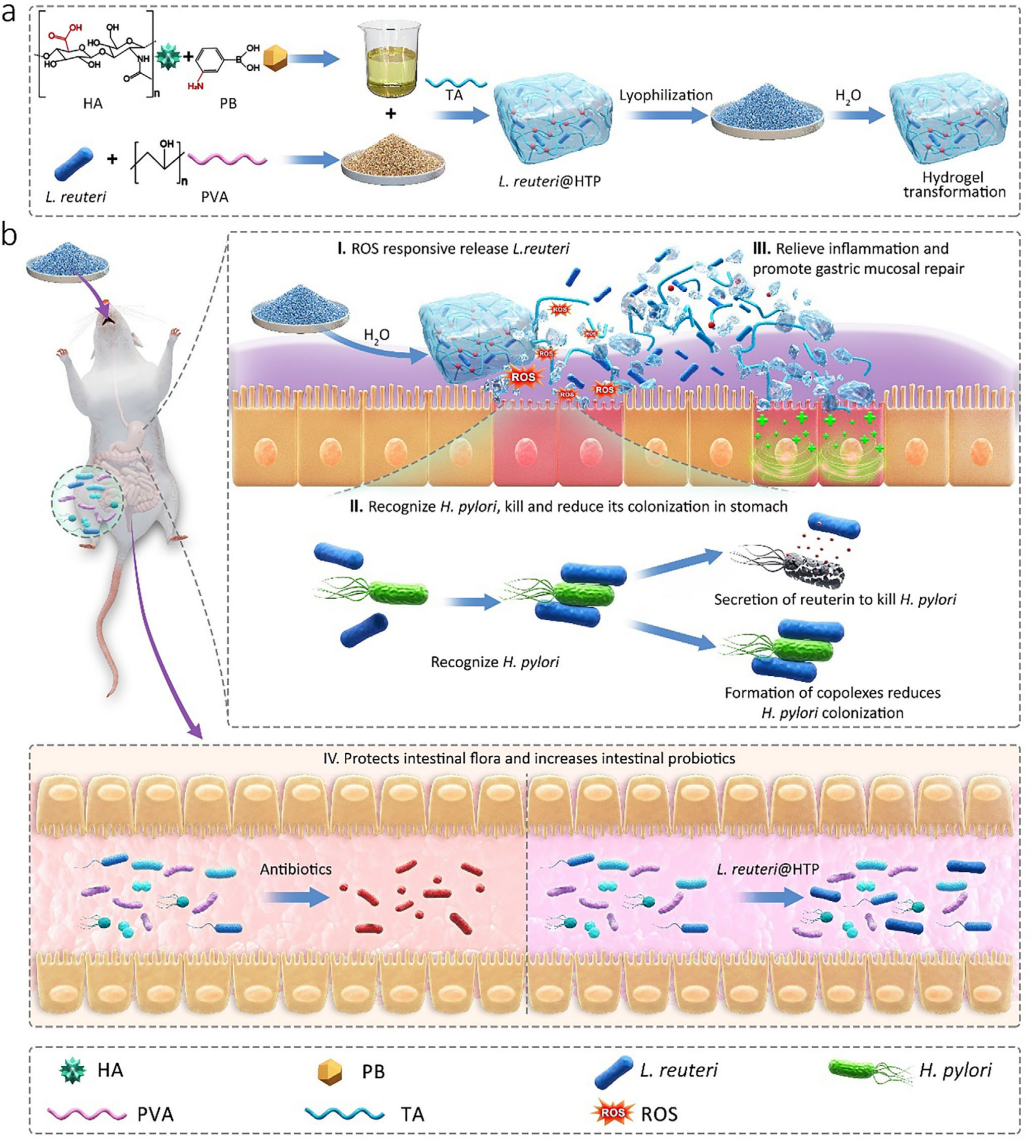
a) The illustration of *L. reuteri*@HTP powder preparation and hydrogel transformation. b) (I) hydrogel transformation of *L. reuteri*@HTP powder in gastric fluid and ROS‐responsive breakdown of transformed *L. reuteri*@HTP hydrogel. (II) *L. reuteri* targets and binds to *H. pylori*, releasing reuterin to inhibit it and forming complexes to reduce gastric colonization. (IV) TA and HA in the hydrogel alleviate inflammation and support gastric mucosa repair. (V) Transformed *L. reuteri*@HTP hydrogel does not disrupt the gut microbiota and promotes the restoration of a healthy intestinal microbiome.

In this study, we designed a novel *H. pylori* eradication strategy based on an edible and hydrogel‐transformable probiotic powder, which is composed of hyaluronic acid (HA) modified with 3‐aminophenylboronic acid (PB) (HA‐PB), tannic acid (TA), polyvinyl alcohol (PVA) and *L. reuteri* (termed *L. reuteri*@HTP, **Scheme** **1**). The *L. reuteri*@HTP powder can rapidly transform into a hydrogel within the gastric environment, thereby enhancing the viability of *L. reuteri* under harsh conditions. It subsequently degrades at inflammatory sites in the stomach, where reactive oxygen species (ROS) are abundant and *H. pylori* aggregate, leading to the on‐target release of *L. reuteri*. The released *L. reuteri* selectively binds to *H. pylori*, reduces *H. pylori* colonization, and secretes reuterin to kill *H. pylori*. On the other hand, TA, which contains multiple phenolic hydroxyl groups, serves as a free radical scavenger, imparting anti‐inflammatory and antioxidant properties to the hydrogel. At the same time, HA facilitates the repair of damaged gastric mucosa.^[^ ^19^ ^]^ Furthermore, *L. reuteri*@HTP powder does not affect gut microbiota homeostasis and promotes the restoration of a healthy gut microbiome. Notably, *L. reuteri*@HTP powder can be stored at room temperature for at least 6 months without compromising the viability of *L. reuteri*. This multifunctional probiotic powder presents a promising candidate for effectively eradicating *H. pylori* in the current context of rising antibiotic resistance.

## 2 Results and Discussion

### 2.1 Preparation and Characterization of the HT and HTP Hydrogel

**Figure 1:**
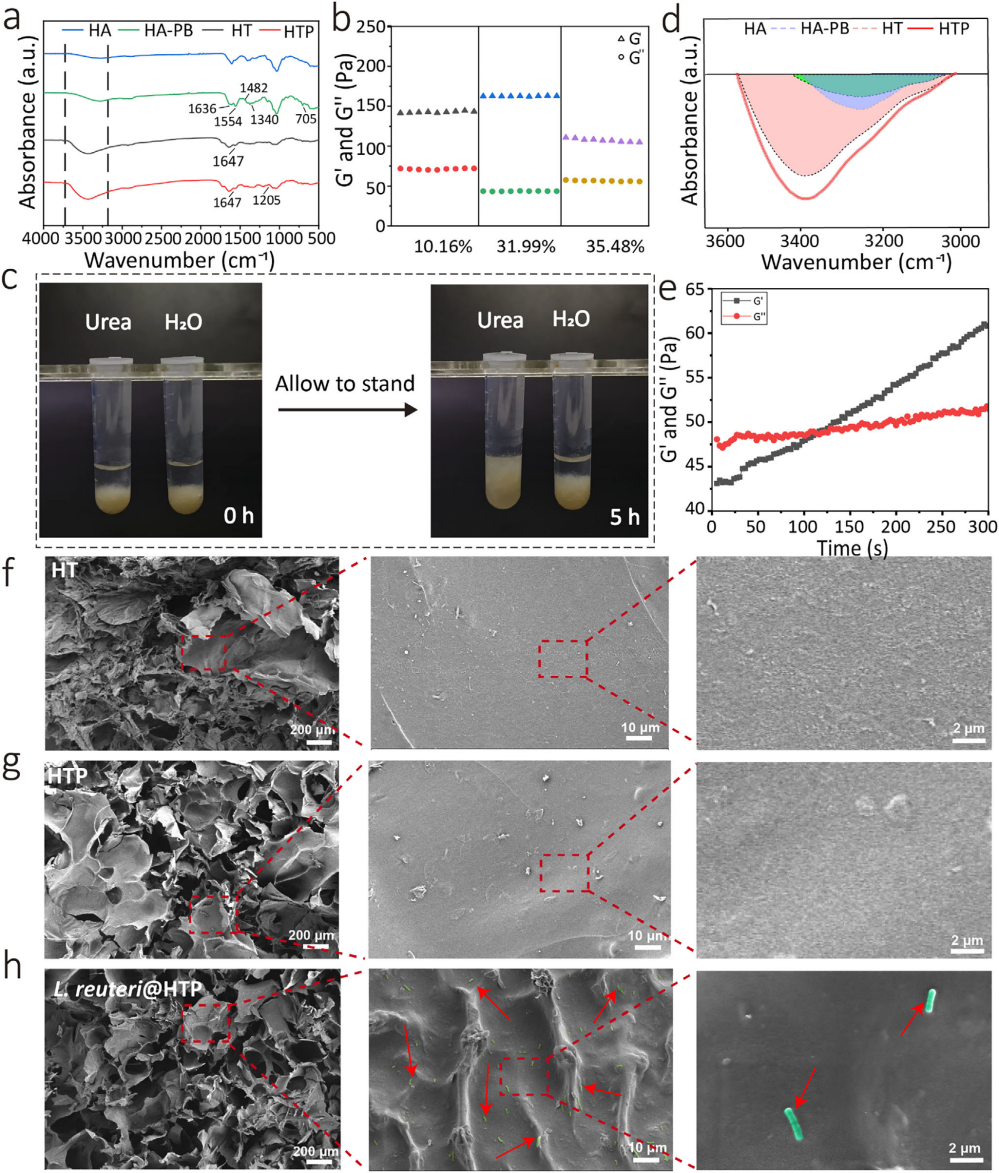
Synthesis and characterization of *L. reuteri*@HTP hydrogel. a) FTIR of HA, HA‐PB, HT hydrogel, and HTP hydrogel. b) Modulus of HT hydrogel. The HA‐PB grafting ratios are 10.16, 31.99, and 35.48%. c) Optical images of *L.reuteri*@HTP hydrogel in urea solution and water. d) FTIR peaks of hydrogen bonds of HA, HA‐PB, HT hydrogel, and HTP hydrogel. e) Dynamic time scanning rheological analysis of *L. reuteri*@HTP hydrogel. SEM images of the cross‐section of (f) HT, g) HTP, and h) *L. reuteri*@HTP hydrogels (*L. reuteri* was marked in green using the Adobe Photoshop (2024 version) software and indicated using red arrows).

HA‐PB was synthesized by activating HA through the EDC/NHS carbodiimide chemistry and subsequently reacted with PB to yield HA‐PB (Figure S1, Supporting Information). In the FTIR spectra, HA‐PB exhibited new peaks at 1636, 1554, and 1482 cm^−1^, corresponding to C═C bending within the phenyl ring. The stretching vibration absorption peak of hydroxyl groups in HT and HTP hydrogels appears at 3395 cm⁻¹ as a broad absorption band. Additional peaks at 1340 cm^−1^ and 705 cm^−1^ were also observed, indicating the stretching and bending vibrations of the phenyl ring and C─H bonds, respectively (**Figure** **1**a). In ^1^H‐NMR, HA‐PB showed multiple peaks at δ = 7.2–7.6 (Figure S2, Supporting Information), which is due to the characteristic peaks generated by the benzene ring on PB. The multiple peaks with δ = 3.3–3.8 are the characteristic peaks of the glycosidic bond of HA, and the position of the peaks did not change before and after grafting. Peaks with δ = 1.9 belong to the methylene peaks. These findings confirm the successful grafting of PB onto HA, with a calculated PB grafting ratio of 31.99%. Upon mixing with TA, the boronic acid groups in HA‐PB rapidly reacted with the catechol groups in TA to form borate ester bonds, producing HT hydrogel. PVA, a water‐soluble polymer rich in hydroxyl groups, imparted good hydrophilicity and hydrogen‐bonding capacity. Therefore, PVA incorporation facilitated the formation of HTP hydrogel, featuring a multi‐network structure with both hydrogen bonds and borate ester bonds (Figure S1, Supporting Information). In this study, we modified HA with PB primarily to introduce boronic ester bonds, thereby enhancing the stability of the hydrogel while enabling its ROS‐responsive degradability.^[^ ^20^ ^]^ In particular, the moduli of the formed hydrogels were successfully adjusted by changing the grafting ratio of PB. Therefore, by controlling the EDC/NHS reaction and stirring time, we prepared HA‐PB with three different grafting ratios—low, medium, and high—and investigated the hydrogel properties under different grafting ratios through rheological analysis. The G' of HT hydrogel with a grafting ratio of 31.99% (161 Pa) was higher than that of both the low grafting ratio group (grafting ratio = 10.16%, G' = 142 Pa) and the high grafting ratio group (grafting ratio = 35.48%, G' = 107 Pa, Figure 1b; Figure S3, Supporting Information). This is primarily because the PB grafting ratio significantly affects the density of borate ester bonds, which in turn influences the gelation properties of the hydrogel and its ability to maintain strength after lyophilization. With an increase in the grafting ratio, the number of reactive sites on HA‐PB available for reaction with TA also increased, resulting in HT hydrogel with a more compact network structure. The decreasing G' curve of the high grafting ratio group indicated poor resistance to deformation and inadequate load‐bearing capacity. Considering the modulus of the gastric mucosa (Young's modulus = 3.27 kPa),^[^ ^21^ ^]^ HA‐PB with a grafting ratio of 31.99% was selected as one of the precursors for the preparation of the HTP hydrogel.

### 2.2 Preparation and Characterization of the *L. reuteri*@HTP Hydrogel

Hydrogen bonds play a critical role in the crosslinked structure of HTP hydrogel, further affecting its capacity to rehydrate and transform into hydrogel from lyophilized powder. To validate this hypothesis, the synthesized HTP hydrogels were individually immersed in urea solution and water. After 5 h of immersion, the hydrogel in the aqueous solution showed minimal structural change, while the hydrogel in the urea solution exhibited pronounced alterations, with a noticeable loosening of the structure (Figure 1c). The absorption peak at 1205 cm⁻¹ further supports the presence of abundant hydrogen bonds in the HTP hydrogel (Figure 1d). It's worth noting that the ─OH FTIR band exhibited a blue shift, moving to a lower wavenumber, suggesting that intermolecular hydrogen bonding reduced the continuity of chemical shifts after the addition of PVA.

To further investigate the hydrogel formation, the molecular weights of HA and the concentrations of TA and PVA were varied. Dynamic time‐scanning rheology results showed that the gelation time was 132 s and the gelation modulus was 22.4 Pa when using low molecular weight HA. In comparison, the high molecular weight (> 1.8 MDa) HA group exhibited a longer gelation time of 183 s, while the medium molecular weight (1.0–1.8 MDa) HA group showed a gelation time of 126 s, indicating that both low and medium molecular weight HAs gelled more rapidly. Regarding gelation modulus, the medium molecular weight HA group achieved the highest value at 28.2 Pa, followed by the high molecular weight group at 26.8 Pa and the low molecular weight group at 22.1 Pa (Figure S4, Supporting Information). This may be attributed to that the HA with low molecular weight has a shorter chain length, which makes it difficult to form a stabilized network. High molecular weight HA has more cross‐linking points, resulting in a higher hydrogel modulus and longer hydrogel formation time.^[^ ^22^ ^]^ Furthermore, as the concentration of TA increased, the cross‐linking speed increased (Figure S5, Supporting Information). The hydrogel modulus was 26.4 Pa (using 300 µL of TA), which was lower than using 200 µL of TA (27 Pa) and higher than using 100 µL of TA (19.6 Pa). The influence of PVA concentration on the hydrogel formation was finally studied. At a lower PVA concentration (i.e., an equivalent of 100 µL), the HTP hydrogel exhibited a longer gelation time (178 s) and lower modulus (23.8 Pa, Figure S6a, Supporting Information). When an equivalent of 200 µL of PVA was added, the gelation time and modulus significantly decreased to 91 s and increased to 46.2 Pa, respectively (Figure 1e). However, further increases in PVA concentration (i.e., an equivalent of 300 µL) did not markedly change the gelation time (124 s) and hydrogel modulus (63 Pa, Figure s6 b, Supporting Information). This is primarily because a high concentration of TA may cause uneven hydrogel formation, resulting in a decrease in the final modulus of the HT hydrogel.^[^ ^23^ ^]^ Based on these results, HA with medium molecular weight was used to crosslink with medium concentrations of TA and PVA in this study.

In addition, *L. reuteri* incorporation did not significantly alter the formation of the hydrogel (Figure S7). Rheological analysis further indicated that replacing PVA with *L. reuteri*@PVA did not affect the gelation time or modulus of the hydrogel (Figure 1e). SEM observations revealed that all the hydrogels exhibited irregular pores. The porosities of HT, HTP, and *L. reuteri*@HTP hydrogels were 41.94, 51.27, and 49.33%, respectively (Figure 1f–h; Figure S8, Supporting Information). SEM observations confirm that *L. reuteri*@HTP displayed a similar porous and sponge‐like structure with HTP. The rod‐shaped structures (highlighted in green, Figure 1h), resembling the morphology of *L. reuteri* bacteria (Figure S9, Supporting Information), confirm the successful encapsulation of *L. reuteri*.

### 2.3 Preparation and Hydrogel Transform Capabilities of *L. reuteri*@HTP Powder

**Figure 2:**
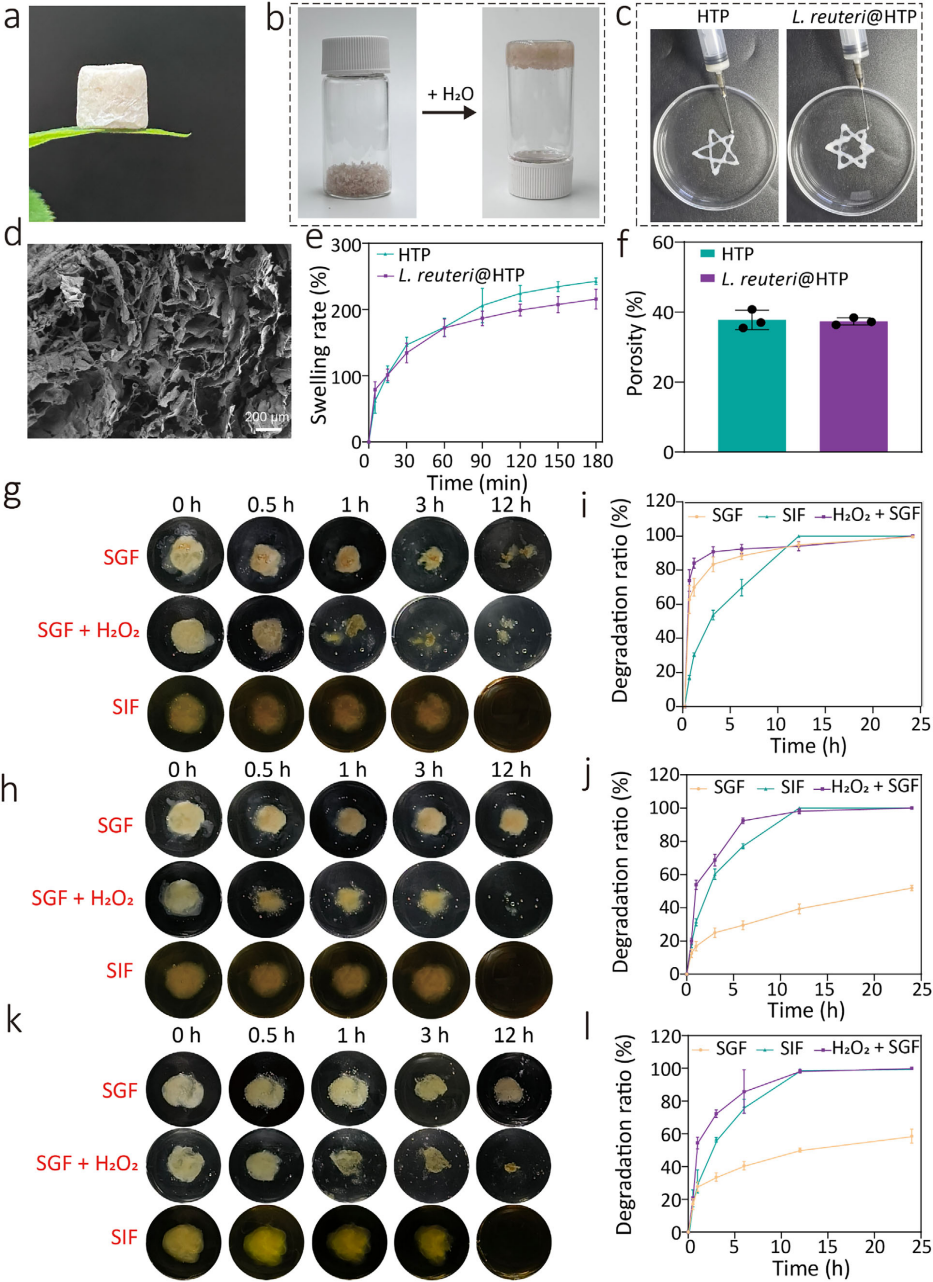
In vitro performance testing of transformed *L. reuteri*@HTP hydrogels. a) The picture of the lyophilized *L. reuteri*@HTP hydrogel. b) *L. reuteri*@HTP powder‐hydrogel transformation process. c) Injectability of HTP and transformed *L. reuteri*@HTP hydrogels. d) SEM image of transformed *L. reuteri*@HTP hydrogels. e) The swelling rate of HTP, and transformed *L. reuteri*@HTP hydrogels. f) The porosity of HTP and transformed *L. reuteri*@HTP hydrogels. g) Digital photographs of HT hydrogels degraded in different environments. h) Digital photographs of HTP hydrogels degraded in different environments. i) Degradation curves of HT hydrogel in different environments. j) Degradation curves of HTP hydrogel in different environments. k) Digital photographs of transformed *L. reuteri*@HTP hydrogel degraded in different environments. l) Degradation curves of transformed *L. reuteri*@HTP hydrogel in different environments. Data are presented as the mean ± SD (n = 3).

Therefore, HA with a molecular weight of 1.0–1.8 MDa and PB grafting ratio of 31.99% was selected for preparing HTP hydrogel for subsequent experiments. The precursor solution composed of HA‐PB (4 mg mL^−1^), PVA (2 mg mL^−1^), and TA (4 mg mL^−1^) with a volume ratio of 3: 1: 1 was used for the following studies. To prepare the powder, hydrogels were lyophilized. By grinding the lyophilized hydrogel, HTP, and *L. reuteri*@HTP powders were obtained, both demonstrating excellent hydrogel transformation capabilities upon contacting water (**Figure** **2**a,b; Figure S10, Supporting Information). The resulting HTP hydrogel demonstrates excellent injectability, and *L. reuteri* does not significantly affect such injectability (Figure 2c). This indicates that the transformed *L. reuteri*@HTP hydrogel exhibits favorable shear‐thinning behavior and fluidity, which contributes to enhancing its adhesion and retention properties in the stomach.^[^ ^24^ ^]^ The rheological curves of the transformed *L. reuteri*@HTP hydrogel showed that the G2 and G″ curves exhibit a parallel trend without intersection, indicating that the transformed hydrogel remains relatively stable and possesses good resistance to compression and deformation (Figure S11, Supporting Information). SEM confirms that the morphology of the transformed *L. reuteri*@HTP hydrogel (porosity = 47.03%) is largely unchanged compared to the original *L. reuteri*@HTP hydrogel (Figure 2d). Swelling behavior studies reveal that swelling rates for HTP and transformed *L. reuteri*@HTP hydrogel were 78.84, and 76.94%, respectively within 5 min. After 2 h, this transformed hydrogel reaches fluid absorption‐swelling equilibrium (Figure 2e). These results indicate no significant difference in the swelling rate between the HTP hydrogel and transformed *L. reuteri*@HTP hydrogel, likely due to their similar porosity (Figure 2f). This finding further confirms that *L. reuteri* does not affect the swelling behavior of the HTP hydrogel.

### 2.4 Degradation Properties of Hydrogels

A defining feature of boronic ester bonds is their sensitivity to ROS, a property anticipated to confer ROS‐responsive degradable characteristics to HT, HTP, and *L. reuteri*@HTP hydrogels. To validate this hypothesis, we immersed HT and HTP hydrogels in simulated gastric fluid (SGF), simulated inflammatory gastric fluid (SGF + 0.1% H_2_O_2_, v/v), and SIF and observed their degradation at various time points. Upon immersion in SGF for 0.5 h, HT hydrogel rapidly disintegrated, with a degradation rate of 62.8 ± 8.2%. After 3 h, the degradation rate reached 83.4 ± 4.3%, and in SGF + H_2_O_2_, the rate was 90.8 ± 3.0% (Figure 2g,i). This rapid degradation may lead to off‐target effects and premature release of probiotics, reducing the therapeutic efficacy of the hydrogel. In contrast, HTP hydrogel degraded more slowly, with degradation rates of 25.0 ± 4.9% and 68.7 ± 3.4% in SGF and SGF + H_2_O_2_, respectively within 3 h (Figure 2h,j). This slower degradation can be attributed primarily to the introduction of hydrogen bonds, which increase the overall crosslinking density of the HTP hydrogel, thereby slowing its degradation rate in solution. Additionally, the transformation process from *L. reuteri*@HTP powder to *L. reuteri*@HTP hydrogel does not alter its degradation behavior (Figure 2k,l). During degradation, TA within the hydrogel rapidly oxidized in the alkaline environment of SIF, causing the solution to turn brownish‐yellow. The above results indicate that the *L. reuteri*@HTP constructed in this study possesses ROS‐responsive degradability. *H. pylori* infection is often associated with gastric inflammation, with inflamed sites typically containing high levels of ROS.^[^ ^25^ ^]^ Therefore, the ROS‐sensitive HTP hydrogel can undergo responsive degradation under ROS conditions, facilitating the targeted release of *L. reuteri* to the *H. pylori* microenvironment for effective eradication. Notably, both HT and HTP hydrogels degraded slowly in SIF, suggesting they can break down gradually in the intestine, thereby exerting minimal interference with normal physiological functions.

### 2.5 *L. reuteri* Viability in HTP Hydrogel and *L. reuteri*@HTP Powder

**Figure 3:**
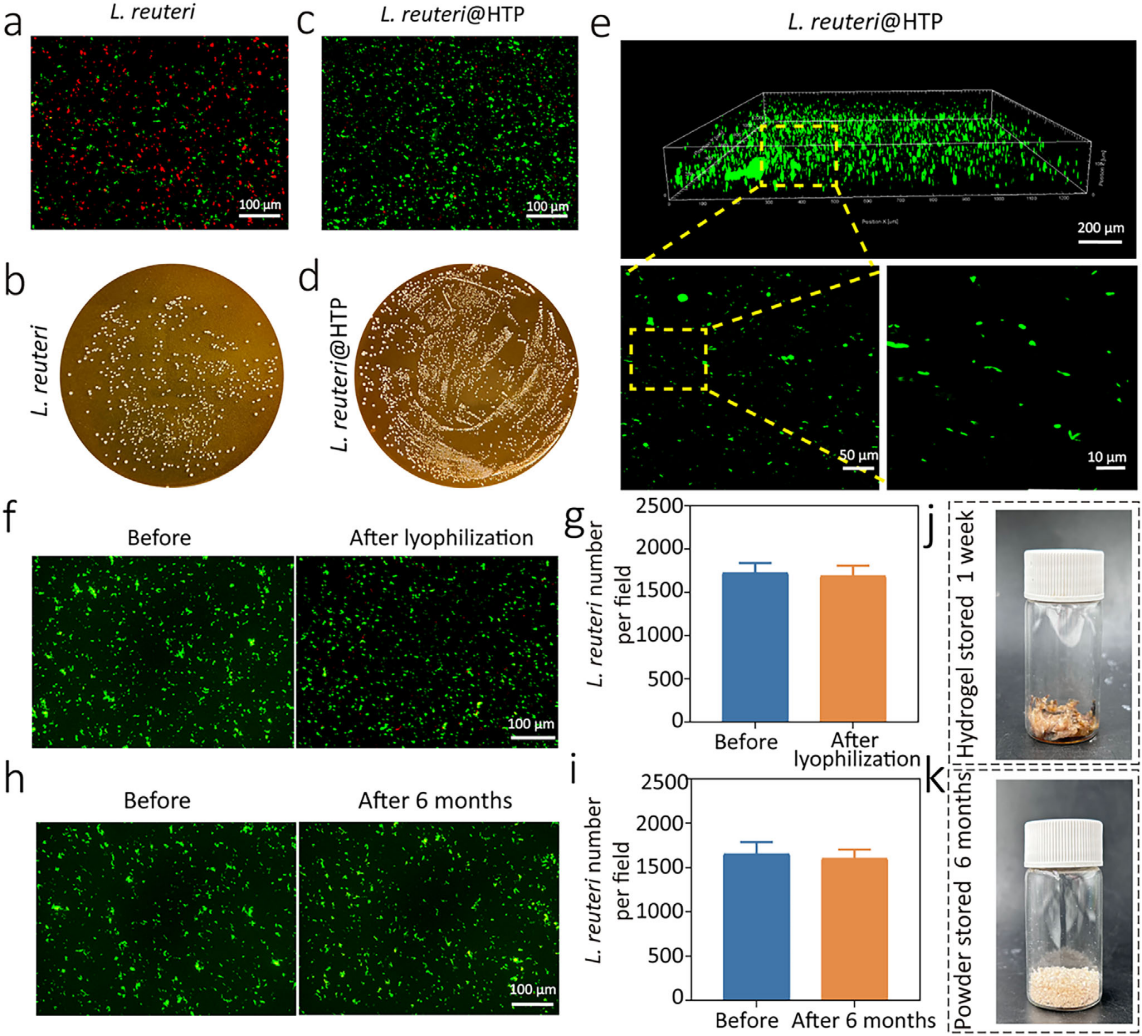
The ability of *L. reuteri*@HTP powder to maintain bacterial viability and extend storage time. a) Live/Dead bacterial staining and (b) colony plate images of *L. reuteri* and transformed *L. reuteri@*HTP hydrogel after coculture with SGF + H_2_O_2_. c) Live/Dead bacterial staining and (d) the quantitative analysis of the viable *L. reuteri* population in *L. reuteri*@HTP hydrogel and *L. reuteri*@HTP power. e) Live/Dead bacterial staining of *L. reuteri* in pristine *L. reuteri*@HTP hydrogel and enlarged views. f) Live/Dead bacterial staining and g) the quantitative analysis of *L.reuteri* before (i.e., in *L. reuteri@*HTP hydrogel) and after lyophilization (i.e., in *L. reuteri@*HTP power). h) Live/Dead bacterial staining and (i) the quantitative analysis of the viable *L. reuteri* population in *L. reuteri*@HTP power before and after 6 months of storage. The appearance of (j) *L. reuteri*@HTP hydrogel after 1 week of storage and (k) *L. reuteri*@HTP powder after 6 months of storage. Data are presented as the mean ± SD (n = 3).

After incubating *L. reuteri* in SGF + H_2_O_2_ for 2 h, the number of viable *L. reuteri* decreased compared to pre‐incubation levels (**Figure** **3**a). Plate coating assays further corroborated the decreased *L. reuteri* activity in SGF + H_2_O_2_ (Figure 3b). This finding suggests that gastric acid affects the vitality of *L. reuteri*. Then, the viability of *L. reuteri* in *L. reuteri*@HTP hydrogel and a simulated gastritis environment was studied, which revealed a significantly higher survival rate for *L. reuteri* in the *L. reuteri*@HTP hydrogel (Figure 3c). Plate coating assays further corroborated that the HTP hydrogel enhanced the *L. reuteri* activity in SGF + H_2_O_2_ (Figure 3d). High‐magnification observations revealed *L. reuteri* as elongated rods, confirming that its viability remained unaffected (Figure 3e). Further quantitative analysis of the bacterial population revealed that when *L. reuteri* was placed into the SGF at an initial concentration of 10⁶ CFU mL^−1^ and incubated for 24 h, only ≈10^2^–10^3^ CFU mL^−1^ remained viable. In contrast, the concentration of *L. reuteri* in the *L. reuteri*@HTP group remained at ≈10⁶ CFU mL^−1^ (Figure S12, Supporting Information). These results suggest that the *L. reuteri*@HTP hydrogel can effectively mitigate the impact of gastric acid on the vitality of *L. reuteri*. The lyophilization process does not affect the viability of *L. reuteri* within the hydrogel, as shown by unchanged viability before and after the process (Figure 3f,g).

Furthermore, the viability of *L. reuteri* in *L. reuteri*@HTP hydrogel and *L. reuteri*@HTP powder after 6‐month storage was evaluated. The Live/Dead bacterial staining results reported no significant change in the *L. reuteri* viability before and after 6 months of storage in a ventilated environment (Figure 3h,i). Moreover, *L. reuteri*@HTP hydrogel underwent visible morphological changes, becoming dried and moldy after 1 week of storage (Figure 3j), whereas the *L. reuteri*@HTP powder retained its original appearance even after 6 months (Figure 3k). The above results validate that *L. reuteri*@HTP powder effectively maintains probiotic viability and exhibits excellent storage stability. This extended protection of *L. reuteri* in *L. reuteri*@HTP powder is largely attributed to the low moisture content of the powder, which significantly minimizes microbial metabolic activity.

In summary, the *L. reuteri*@HTP powder developed in this study demonstrates significant potential for clinical translation. First, our findings confirm that *L. reuteri*@HTP powder ensures long‐term storage stability while preserving the viability of *L. reuteri*, facilitating large‐scale production and distribution. Second, its ability to retransform to hydrogel in the gastric environment not only effectively protects *L. reuteri* from gastric acid degradation but also enhances its retention within the gastric mucosa, thereby improving its delivery efficiency. These properties are crucial for maintaining the bioactivity of *L. reuteri* and prolonging its therapeutic effects.

### 2.6 In Vitro Biocompatibility of *L. reuteri*@HTP Powder

**Figure 4:**
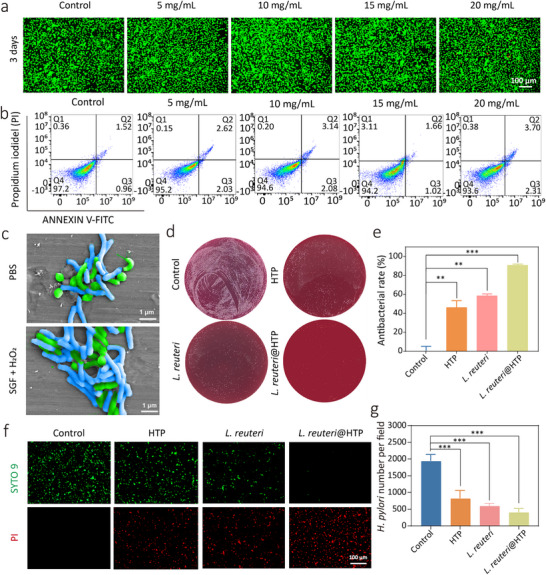
Cellular safety and in vitro antibacterial activity of *L. reuteri*@HTP powder. a) Live/Dead cell staining of HFE‐145 cells after coculture with *L. reuteri*@HTP powder with different concentrations for 3 days. b) Flow cytometry analyzes the apoptosis rate of HFE‐145 cells after co‐culturing with *L. reuteri*@HTP powder with different concentrations (5, 10, 15, and 20 mg mL^−1^) for 1 day. c) The targeted binding ability of *L. reuteri* to *H. pylori* in PBS and simulated gastritis environment (SGF + H_2_O_2_), *L. reuteri*: blue, appears as short rod‐shaped or bacillary forms with a smooth surface, blunt‐rounded ends, no significant curvature, and no flagella; *H. pylori*: green, exhibits a characteristic helical or curved shape, with some strains appearing as slightly curved rods. Its surface is rough, and its tail end is often equipped with flagella. d) Colony plate images of different samples after co‐culturing with *H. pylori* in vitro. e) The corresponding antibacterial rate after different treatments. f) Live/Dead bacterial staining of *H. pylori* after different treatments. g) The quantitative analysis of live *H. pylori* after different treatments. Data are presented as the mean ± SD (n = 3), ^*^ *p* < 0.05, ^**^ *p* < 0.01, and ^***^ *p* < 0.001.

Biocompatibility is a critical factor for the clinical translation of biomaterials. To evaluate the cytotoxicity of *L. reuteri*@HTP powder, *L. reuteri*@HTP powder was transformed into a hydrogel in a cell culture medium and subsequently co‐cultured with HFE‐145 cells. The results showed that HFE‐145 cells maintained high viability (> 85%) across different *L. reuteri*@HTP powder concentrations over 3 days (**Figure** **4**a; Figures S13–S15, Supporting Information), indicating minimal cytotoxicity of *L. reuteri*@HTP powder. Flow cytometry further confirmed that the proportion of apoptotic cells did not significantly increase following treatment at different *L. reuteri*@HTP powder concentrations compared to the control (Figure 4b). To assess its blood compatibility, a hemolysis assay was conducted, using PBS as a negative control and water as a positive control. Results demonstrate that coincubation with *L. reuteri*@HTP powder at varying concentrations does not induce significant hemolysis in rat red blood cells. Even at 20 mg mL^−1^, the hemolysis rate remained below 5%, indicating excellent blood compatibility of *L. reuteri*@HTP powder (Figure S16, Supporting Information). Collectively, these results indicate the favorable in vitro biocompatibility of *L. reuteri*@HTP powder.

### 2.7 In Vitro Anti‐*H. pylori* Ability of *L. reuteri*@HTP Powder

*L. reuteri*—a common gut probiotic in mammals—can interact with *H. pylori* through specific surface interactions.^[^ ^16^, ^32^ ^]^ The formed *L. reuteri‐H. pylori* aggregates are then naturally expelled from the stomach. Moreover, *L. reuteri* has been reported to inhibit harmful bacteria, such as *Escherichia coli* and *Salmonella*, by secreting reuterin,^[^ ^26^ ^]^ thus reducing *H. pylori* colonization. We cultured *L. reuteri* and *H. pylori* in a simulated gastritis environment (SGF + H_2_O_2_) for 2 h. SEM imaging confirmed that *L. reuteri* (blue, appears as short rod‐shaped or bacillary forms with a smooth surface, blunt‐rounded ends, no significant curvature, and no flagella) maintained its ability to bind and form aggregates with *H. pylori* (green, exhibits a characteristic helical or curved shape, with some strains appearing as slightly curved rods. Its surface is rough, and its tail end is often equipped with flagella) under these conditions, similar to the PBS environment (Figure 4c). This finding suggests that *L. reuteri* retains its targeting and binding capacity even under gastric acidity and inflammation conditions, highlighting the in vivo potential of *L. reuteri*@HTP powder to target *H. pylori*. To evaluate whether encapsulation in HTP impacts its bactericidal effect on *H. pylori*, *L. reuteri*, HTP powder, and *L. reuteri@HTP* powder were co‐cultured with *H. pylori* for 24 h. Plate assays revealed that while HTP reduced *H. pylori* growth, *L. reuteri* led to an even greater decrease, and *L. reuteri@HTP* powder inhibited over 90% of *H. pylori* (Figure 4d,e). Additionally, supernatants from *L. reuteri* cultures showed a significant increase in dead *H. pylori*, with *L. reuteri@HTP* powder displaying the highest antibacterial activity (Figure 4f,g). *L. reuteri* also exhibited a certain degree of bactericidal activity, primarily attributed to its secretion of reuterin, an antimicrobial compound.^[^ ^26a^ ^]^ HTP demonstrated some antibacterial activity as well, mainly due to the presence of TA in HTP.^[^ ^27^ ^]^ Notably, *L. reuteri*@HTP exhibited the strongest bactericidal effect, likely because HTP encapsulation significantly enhanced the viability of *L. reuteri*. Additionally, HTP encapsulation prolonged the release of reuterin, allowing it to exert a sustained antibacterial effect against *H. pylori*. This extended duration of action substantially improved the antibacterial efficacy of *L. reuteri*.

### 2.8 In Vitro Free Radical Scavenging and Mucosal Repair Ability of *L. reuteri*@HTP Powder

*H. pylori* secrete CagA and VacA toxins, which recruit immune cells and induce the excessive production of free radicals.^[^ ^28^ ^]^ These free radicals may impair the bactericidal efficiency against *H. pylori* and potentially contribute to the development of other diseases.^[^ ^29^ ^]^ Therefore, the effective removal of excessive ROS is crucial for the eradication of *H. pylori*. DPPH· and PTIO· free radical scavenging assays were conducted to evaluate the RNS and ROS scavenging ability of *L. reuteri*@HTP powder, respectively. The color of the DPPH· radical solution rapidly decreased over time, exhibiting a clearance rate of 31.7 ± 2.5% at 30 s, 90.9 ± 1.2% at 90 s, and a peak clearance rate of 94.7 ± 0.4% for DPPH· radicals at 180 s (Figure S17a, Supporting Information). For PTIO· radical solution, its color faded rapidly over time (Figure S17b, Supporting Information), indicating a gradual increase in ROS clearance by the *L. reuteri*@HTP powder. Quantitative analysis revealed that the clearance rate of ROS by the *L. reuteri*@HTP powder reached 59.9 ± 2.4% at 120 min. The RNS and ROS scavenging ability is primarily due to the strong electron‐donating capacity of TA, which donates electrons to free radicals such as DPPH· and PTIO.^[^ ^19a^ ^]^

**Figure 5:**
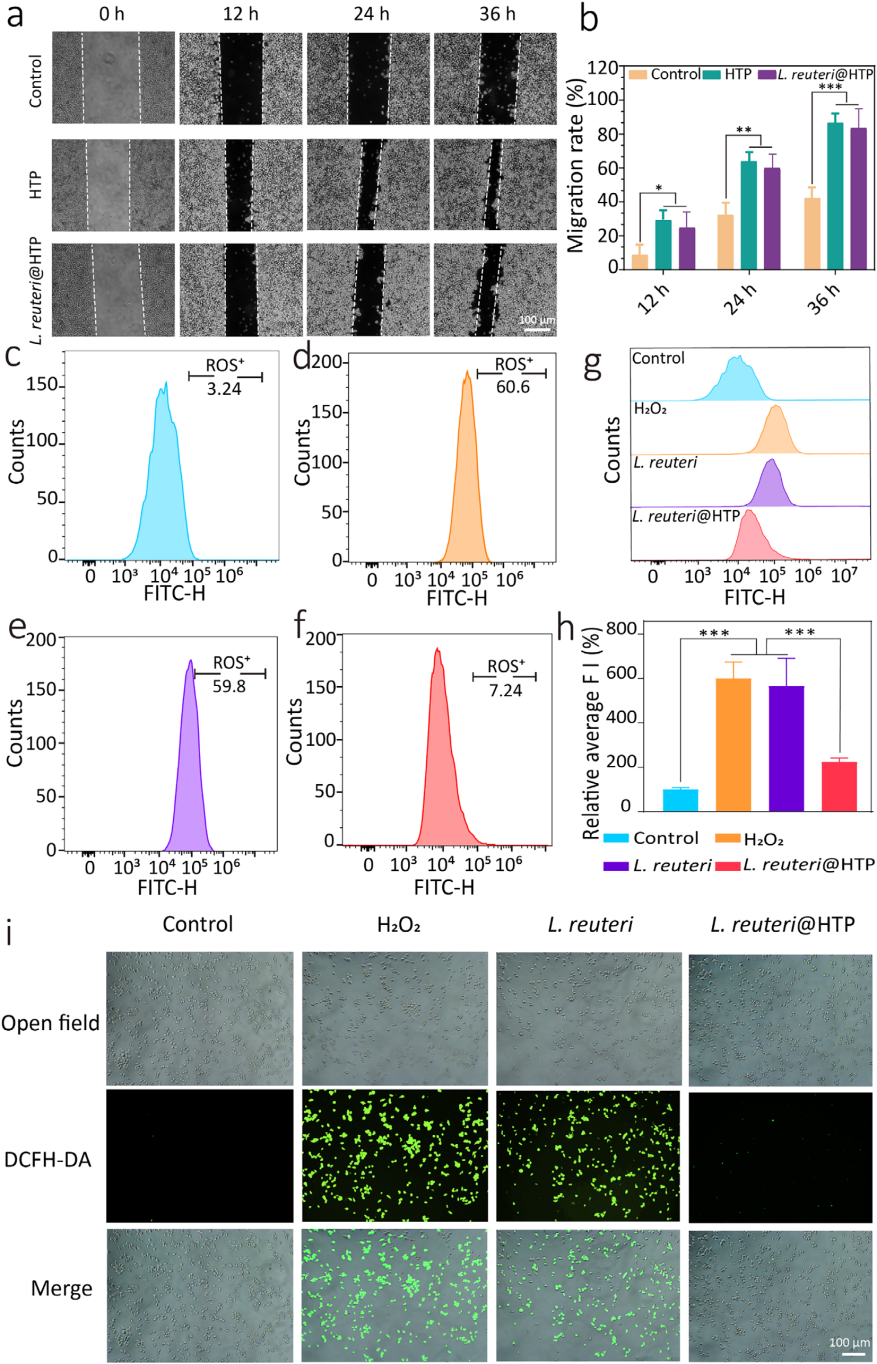
In vitro cell proliferation promotion and ROS clearance by *L. reuteri*@HTP powder. a) The cell scratch picture at different time points post‐treatment. b) The quantitative analysis of wound closure at 12, 24, and 36 h. Quantitative analysis of ROS levels in RAW264.7 cells in (c) control group, d) H_2_O_2_ group, e) *L. reuteri* group, and f) *L. reuteri*@HTP group. g) Mountain range plot showing ROS content in RAW264.7 cells after different treatments. h) Relative average fluorescence intensity of flow cytometry after different treatments. i) The fluorescence images of HFE‐145 cells following various treatments. Data are presented as the mean ± SD (n = 3), ^*^ *p* < 0.05, ^**^ *p* < 0.01, and ^***^ *p* < 0.001.

Besides, *H. pylori* infection is usually associated with abnormalities in gastric acid secretion, which subsequently lead to gastric mucosal damage.^[^ ^30^ ^]^ Thus, addressing gastric mucosal damage and inflammation is also essential for the effective treatment of *H. pylori* infection. A cell scratch assay was carried out to evaluate the cell proliferation‐promoting effect of *L. reuteri*@HTP powder. The *L. reuteri*@HTP powder group demonstrated a significant increase in cell migration than the control group (**Figure** **5**a,b). This enhancement is primarily due to that the HA facilitates the expression of adhesion molecules. Furthermore, HA can interact with cell surface receptors (such as CD44 and RHAMM) and activate intracellular signaling pathways to promote cell proliferation and differentiation.^[^ ^31^ ^]^ The HTP group demonstrates similar cell migration rates compared to the *L. reuteri*@HTP powder treatment group, suggesting that *L. reuteri* does not compromise its ability to promote cell proliferation. These findings indicate that *L. reuteri*@HTP powder has significant potential for enhancing gastric mucosal repair.

### 2.9 *Intra‐Cellular* Free Radical Scavenging Ability of *L. reuteri*@HTP Powder

To investigate whether *L. reuteri*@HTP powder can mitigate cellular free radicals, we treated RAW264.7 cells with H_2_O_2_ to establish a cellular inflammation model. Subsequently, the cells were treated with PBS, *L. reuteri*, and *L. reuteri*@HTP powder, respectively. DCFH‐DA was utilized as a probe to label ROS and measure the ROS levels in the cells through flow cytometry and cell fluorescence. The flow cytometry results indicated that, compared to the cells treated with H_2_O_2_ alone, there was no significant change in the proportion of ROS‐positive cells following treatment with *L. reuteri*. In contrast, treatment with *L. reuteri*@HTP powder resulted in a substantial decrease in the number of ROS‐positive cells from 60.6 to 7.24%, demonstrating the excellent ROS scavenging ability of *L. reuteri*@HTP powder (Figure 5c–h). Furthermore, fluorescence microscopy revealed a marked reduction in green fluorescence in the *L. reuteri*@HTP powder treatment group compared to the positive control group (Figure 5i). These results collectively demonstrate that *L. reuteri*@HTP powder has a significant capacity to clear intracellular ROS, highlighting its potential to mitigate oxidative stress‐related damage.

### 2.10 In Vivo Biocompatibility of *L. reuteri*@HTP Powder

*L. reuteri*@HTP powder can transform into a hydrogel in the stomach and adhere to the gastric mucosa, facilitating its therapeutic effects. Once attached to the gastric mucosa, it may interact with host tissues, making it crucial to assess its biocompatibility. Mice were treated with PBS, HTP hydrogel, *L. reuteri*, antibiotics, or the *L. reuteri*@HTP powder once daily for one week. The heart, liver, spleen, lung, and kidney were then sliced for H&E staining. Compared to those in the control group (healthy mice), no obvious signs of toxicity or inflammation were observed in any of the treated mice (Figure S18, Supporting Information). Additionally, routine blood and blood biochemistry tests indicate no abnormal routine blood and biochemical parameter fluctuation relative to the control group (Figures S19 and S20, Supporting Information). These findings indicate that *L. reuteri*@HTP powder demonstrates outstanding in vivo biocompatibility.

### 2.11 In Vivo Anti‐*H. Pylori* Capacity of *L. reuteri*@HTP Powder

**Figure 6:**
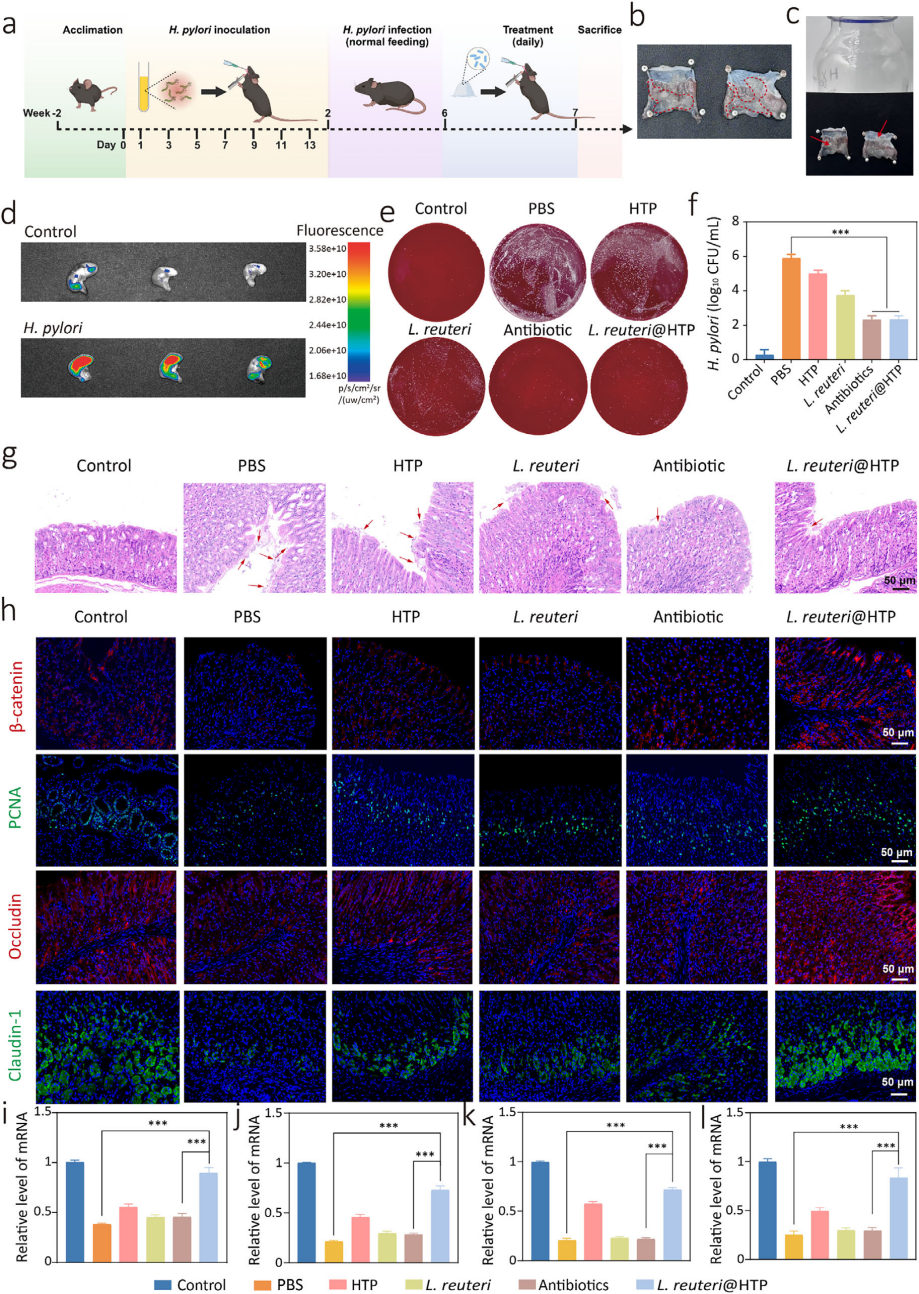
In vivo antibacterial and gastric mucosal repair capabilities of *L. reuteri*@HTP powder. a) Flowchart of the construction and treatment process for the *H. pylori*‐infected mouse model. b) The picture represents that HTP powder can transform into a hydrogel in the stomach of mice. c) Appealing the intragastric hydrogel upright, the hydrogel can be found to adhere to the stomach. d) The IVIS imaging pictures of gastric tissues in *H. pylori*‐infected and healthy mice. e) Digital photographs of blood agar plate cultures of *H. pylori*‐infected mice after different treatments. f) The corresponding *H. pylori* colony counting plots after different treatments. g) H&E staining of mouse gastric tissue to evaluate the *H. pylor*i‐infected situation after different treatments (red arrows represent *H. pylori*). h) Immunofluorescence staining of HEF‐145 cells after different treatments. i–l) mRNA expression levels of (i) β‐catenin, (j) PCNA, (k) Occludin, and (l) Claudin‐1 in gastric tissue following different treatment regimens. Data are presented as the mean ± SD (n = 3), ^*^ *p* < 0.05, ^**^ *p* < 0.01, and ^***^ *p* < 0.001.

To evaluate the in vivo efficacy of *L. reuteri*@HTP powder in clearing *H. pylori*, *H. pylori*‐infected C57BL/6 mice models were established, as outlined in **Figure** **6**a. The success of model construction was confirmed by rapid urease testing, plate streaking, and H&E staining on gastric tissue. Compared to the control group, a large number of *H. pylori* colonies appeared in the plate cultures of gastric tissue from the *H. pylori*‐infected mice. H&E staining revealed the presence of numerous curved rod‐shaped bacteria on the gastric mucosa Additionally, the *H. pylori*‐infected group rapid urease tested positive (pink) compared to the control (yellow) (Figure S21, Supporting Information). These results confirm the successful construction of the *H. pylori* animal model. The in vivo hydrogel transition capability of *L. reuteri*@HTP powder was first evaluated. By injecting *L. reuteri*@HTP powder into the mouse stomach using a syringe, a hydrogel was observed on the stomach wall after 1 h (Figure 6b), indicating that the powder could rehydrate and transform into a hydrogel upon contact with water in vivo. More importantly, the transformed hydrogel adhered closely to the gastric mucosa (Figure 6c), thereby prolonging the contact time between *L. reuteri* and *H. pylori* in the stomach, which could potentially enhance the therapeutic efficacy against *H. pylori* infection. To this end, we investigated the gastric retention of *L. reuteri*@HTP powder after it entered into the mouse stomach. We labeled HTP with Cy7 and then encapsulated *L. reuteri* to prepare Cy7‐labeled *L. reuteri*@HTP. The formulation was administered to *H. pylori*‐infected mice via oral gavage, and the results showed that *L. reuteri*@HTP powder could remain in the stomach for at least 2 h (Figure S22, Supporting Information). The gastric inflammation targeting ability of *L. reuteri*@HTP powder was then assessed. Infected and healthy mice were orally administered CY7‐labeled *L. reuteri*@HTP powder and sacrificed after 8 h. IVIS imaging revealed strong fluorescence signals in the gastric tissues of *H. pylori*‐infected mice, with significant fluorescent residue. However, control mice showed significantly lower fluorescence (*p* < 0.001) (Figure 6d; Figure S23, Supporting Information), confirming the targeted release capability of *L. reuteri@HTP* in the inflamed gastric environment. Together with its ROS‐responsive capacity, *L. reuteri*@HTP powder can effectively target and release *L. reuteri* at the site of inflammation. Since gastric inflammation sites often harbor a high concentration of *H. pylori*,^[^ ^14b^ ^]^ this mechanism significantly shortens the time *L. reuteri* takes to reach and interact with *H. pylori* upon entering the stomach, reducing bacterial loss caused by the difficulty of locating *H. pylori* when *L. reuteri* is administered alone. By enhancing the survival rate of *L. reuteri* in the gastric environment and facilitating its targeted interaction with *H. pylori*, *L. reuteri*@HTP greatly improves the efficacy of *L. reuteri* in eliminating *H. pylori* in the stomach.

Subsequently, *H. pylori*‐infected and control mice were treated daily for one week, after which they were sacrificed. Plate coating results showed a decrease in *H. pylori* colonies in the *L. reuteri* treatment group, with the antibiotic‐comparable colony count in the *L. reuteri*@HTP powder group (Figure 6e,f). This suggests that *L. reuteri*@HTP powder effectively eradicates *H. pylori* infection in vivo. H&E staining of gastric tissues revealed numerous *H. pylori* near the gastric mucosa and glands in PBS‐treated infected mice (red arrows). After *L. reuteri* treatment, *H. pylori* count decreased, with further reductions observed following *L. reuteri*@HTP powder treatment. Moreover, the *L. reuteri*@HTP powder treatment group showed no significant difference from antibiotic‐treated mice (Figure 6g). Such an antibiotic‐comparable *H. pylori* eradication efficacy is attributed to HTP encapsulation, which enhances *L. reuteri* survival in the gastric environment. In addition, HTP's responsive release at the site of inflammation improves the bioavailability of *L. reuteri*, thereby enhancing its anti‐*H. pylori* activity.

### 2.12 In Vivo Mucosal Repair Capacity of *L. reuteri*@HTP Powder

To evaluate the repair of the gastric mucosa in mice after treatment with *L. reuteri*@HTP powder, we conducted immunofluorescence analysis of β‐catenin, PCNA, Occludin, and Claudin‐1 expression levels in mouse gastric tissues. β‐catenin and PCNA play crucial roles in regulating cell adhesion, interactions, and communication for the proliferation of HEF‐145 cells.^[^ ^32^ ^]^ Occludin and claudin‐1, which are tight junction proteins in HEF‐145 cells, reflect the repair capacity of the gastric mucosa.^[^ ^33^ ^]^ Increases in their levels indicate an ongoing repair process within the gastric mucosa. The results indicate that *H. pylori* infection reduces protein expression levels by disrupting intercellular adhesion. Upon treatment with HTP, *L. reuteri*, antibiotics, and *L. reuteri*@HTP powder, there was a notable increase in the expression of cell adhesion proteins. Notably, the *L. reuteri*@HTP powder group exhibited the most substantial elevation in these expression levels (Figure 6h; Figure S24, Supporting Information). qPCR analysis confirmed that the mRNA levels of proteins such as β‐catenin, PCNA, Occludin, and Claudin‐1 correlated with their respective protein expression patterns (Figure 6i–l). More interestingly, the gene expression profile following *L. reuteri*@HTP powder treatment closely approximated that of normal gastric epithelial cells, with statistically significant differences observed when compared to other treatment modalities (p < 0.05). These findings suggest that treatment with *L. reuteri*@HTP powder may enhance the expression of adhesion proteins, thereby promoting tight cellular connections and upregulating cell proliferation. Furthermore, treatment with *L. reuteri*@HTP powder improves the structure integrity of tight junction in the gastric mucosa, underscoring its role in restoring and maintaining gastric mucosal barrier function.

### 2.13 In Vivo Anti‐Inflammatory Capacity of *L. reuteri*@HTP Powder

**Figure 7:**
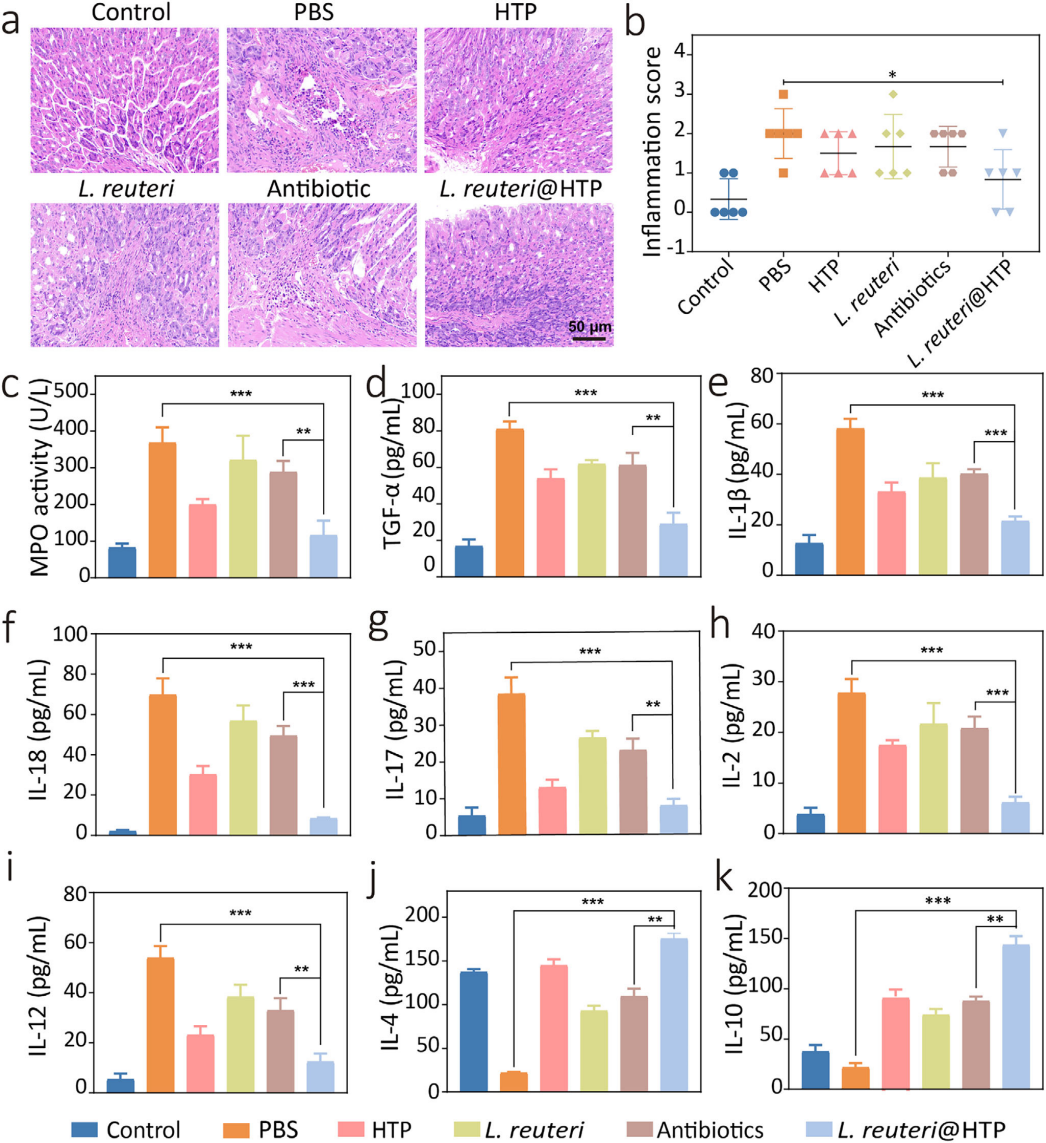
In vivo anti‐inflammatory evaluation of *L. reuteri*@HTP powder. a) H&E staining of mouse gastric tissue following various treatments. b) Inflammation was assessed using the Sydney system, concerning the H&E‐stained images presented in Figure 7a. c) MPO activity, d) TGF‐α, e) IL‐1β, f) IL‐18, g) IL‐17, h) IL‐2, i) IL‐12, j) IL‐4, and k) IL‐10 levels in the serum of treated mice. Data are presented as the mean ± SD (n = 6), ^*^ *p* < 0.05, ^**^ *p* < 0.01, and ^***^ *p* < 0.001.

Subsequently, we investigated the in vivo anti‐inflammatory capacity of *L. reuteri*@HTP powder. H&E staining of gastric tissues from *H. pylori*‐infected mice revealed significant infiltration of inflammatory cells throughout the mucosa (**Figure** **7**a). Following individual treatment with HTP, *L. reuteri*, and antibiotics, a relative reduction in inflammatory cell presence was observed, although some infiltration persisted in the muscularis mucosa. In contrast, treatment with *L. reuteri*@HTP powder resulted in minimal inflammatory cell infiltration in the gastric tissues. These results demonstrate that *L. reuteri*@HTP powder exhibits optimal efficacy in anti‐inflammatory activity, likely attributed to the ROS scavenging capability of TA. Evaluation using the Sydney system indicated that inflammation was nearly absent in the gastric tissues of *L. reuteri*@HTP powder‐treated mice, with results comparable to the control group (Figure 7b). Additionally, the expression of inflammatory cytokines in mice was measured post‐treatment using ELISA. As depicted in Figure 7c–i, treatment with HTP, *L. reuteri*, and antibiotics reduced the expression of proinflammatory cytokines including MPO, TGF‐α, IL‐1β, IL‐18, IL‐17, IL‐2, and IL‐12. Notably, the *L. reuteri*@HTP powder treatment led to the most significant reductions in the levels of these cytokines. Moreover, the *L. reuteri*@HTP powder group exhibits the highest expression levels of the anti‐inflammatory cytokines IL‐4 and IL‐10 (Figure 7j,k). These findings suggest that *L. reuteri*@HTP powder exhibits superior anti‐inflammatory effects. It is noteworthy that *L. reuteri*@HTP powder displays enhanced anti‐inflammatory properties compared to HTP, potentially because its effective *H. pylori* eradication simultaneously enhanced the anti‐inflammatory effects.

The safety concerns associated with prolonged probiotic retention in the stomach are also a critical consideration in clinical translation.^[^ ^34^ ^]^ Our findings indicate that *L. reuteri*@HTP powder exhibits inflammation‐responsive degradability, enabling targeted probiotic release in the presence of gastric inflammation. This strategy significantly minimizes *L. reuteri* release in individuals without *H. pylori* colonization, thereby reducing potential disruptions to the gastric microbiome in healthy individuals. Furthermore, animal experiments demonstrated that the administration of *L. reuteri*@HTP significantly alleviated gastric inflammation in mice. Therefore, the *L. reuteri*@HTP powder effectively mitigates safety concerns associated with prolonged probiotic retention in the stomach, enhancing its clinical applicability.

### 2.14 Effect on the Gut Microbiota of *L. reuteri*@HTP Powder

**Figure 8:**
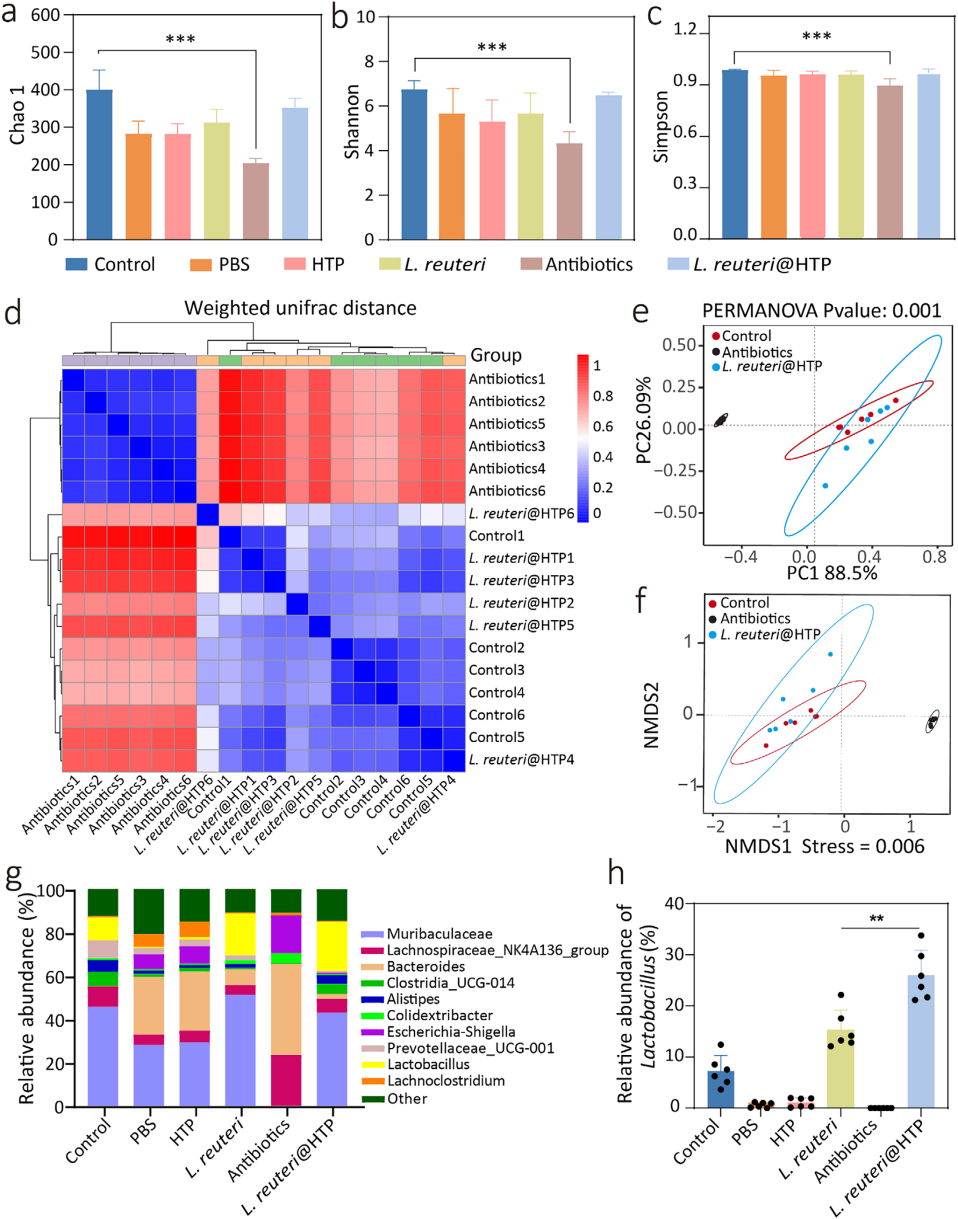
Impact of *L. reuteri*@HTP powder on gut microbiota. a) Chao 1 diversity, b) Shannon indexes, and c) Simpson of α‐diversity in the feces of mice after different treatments. d) The β‐diversity indices of mouse fecal samples under different treatments (blue: closer similarity. red: greater differences). e) PCoA and (f) NMDS of β‐diversity in the feces of mice after different treatments. g) The relative abundance of microbial communities in mouse feces following different treatments. h) The relative abundance of *Lactobacillus* in mouse feces following different treatments. Data are presented as the mean ± SD (n = 6), ^*^ *p* < 0.05, ^**^ *p* < 0.01, and ^***^ *p* < 0.001.

As the second set of genetic systems in the human body, the gut microbiota is intricately associated with many diseases.^[^ ^35^ ^]^ To elucidate the effects of *L. reuteri*@HTP powder treatment on the host gut microbiota, we collected fresh feces from mice post‐treatment and performed 16S rRNA gene sequencing. The results showed that following *H. pylori* infection, the Chao1 index decreased compared to healthy control, which is similar to the previous study.^[^ ^2^ ^]^ However, treatment with HTP or *L. reuteri* led to meaningless improvements in gut microbial diversity (**Figure** **8**a). The Chao1 index significantly decreased in the antibiotic‐treated group compared to the control group (*p* < 0.001). However, in the *L. reuteri*@HTP powder treatment group, the Chao1 index was notably higher than that in the antibiotic‐treated group and *H. pylori*‐infected group, and comparable to the healthy group (*p* > 0.05, Figure 8a). Similar trends were observed in other α diversity metrics, including the Shannon and Simpson indices (Figure 8b,c). These findings indicate that *L. reuteri*@HTP powder treatment has a significantly lower impact on gut microbiota α diversity than antibiotics. Furthermore, a heatmap was used to depict the gut microbiota β diversity among the control group, antibiotic group, and *L. reuteri*@HTP powder group. The β diversity index of the *L. reuteri*@HTP group is nearly identical to that of the control group and significantly distinct from that of the antibiotic group (Figure 8d). PCoA and NMDS analyses of β diversity further confirmed that the β diversity in the *L. reuteri*@HTP powder treatment group is comparable to the healthy group, and exhibited a significant difference compared to the antibiotic group (Figure 8e,f). These findings suggest that *L. reuteri*@HTP powder treatment exerts a milder impact on β diversity than antibiotics and may help partially restore the α diversity disrupted by *H. pylori* infection.

Finally, the relative abundance of bacteria species was examined. *H. pylori* infection induced significant alterations in gut microbiota composition. The most significant adverse changes were observed in the antibiotic treatment group, characterized by a reduction in species diversity and an increase in harmful bacteria, such as *Escherichia–Shigella* and *Colidextribacter*. HTP powder group reports no significant reverse of this negative trend. In contrast, *L. reuteri* treatment produced substantial improvement in the gut microbiota composition, particularly an increase in *Lactobacillus*, a beneficial bacterium widely used in yogurt products and probiotic medications. The bacterial composition and abundance of probiotics in the *L. reuteri@*HTP powder treatment group showed more pronounced improvements and even restored to the levels observed in healthy mice. These findings suggest that *L. reuteri*@HTP powder does not disrupt the gut microbiota and helps restore a healthy microbiome of mice (Figure 8g,h). This is likely attributable to the beneficial effects of *L. reuteri*, which increases butyrate levels in the gut and supports gut homeostasis. In addition, as a lactic acid bacterium, *L. reuteri* synthesizes various enzymes, such as lactic acid, lipase, and bile salt hydrolase, in the gastrointestinal tract, facilitating pH balance and inhibiting bacteria.^[^ ^36^ ^]^

## 3 Conclusion

This study presents an edible polymer‐based probiotic powder, *L. reuteri*@HTP, designed to form a hydrogel in situ within the stomach. This strategy, based on bacteria‐mediated bacterial eradication, aims to improve *H. pylori* treatment while potentially offering additional benefits such as anti‐inflammatory effects, gastric mucosal repair, and microbiota preservation. The powder, utilizing hydrogen bonding and reversible dynamic boronate ester bonds, reconstitutes into a hydrogel in the gastric environment, which extends *L. reuteri*’s storage stability and enhances its survival under acidic conditions. Upon gastric entry, the *L. reuteri*@HTP hydrogel selectively releases *L. reuteri* and its bioactive components, including TA and HA, at inflammation sites. *L. reuteri* targets *H. pylori*, secreting reuterin with bactericidal activity, while TA scavenges ROS and neutralizes pro‐inflammatory agents, and HA promotes gastric mucosal healing by enhancing cell proliferation and adhesion protein expression. Additionally, our findings suggest that *L. reuteri*@HTP treatment may support gut microbiota restoration. Overall, *L. reuteri*@HTP powder demonstrates potential as a supportive approach for *H. pylori* management.

## Ethics Statement

All animal procedures in this study were approved by the Ethics Committee of the First Affiliated Hospital of Naval Medical University (Shanghai, China, Approval Number: CHFC(A.E)2023‐017).

[Correction added on 18 April 2025, after first online publication: Ethics Statement is added.]

## Conflict of Interest

The authors declare no conflict of interest.

## Author Contributions

Y.L. and H.S. contributed equally to this work. Y.L. performed in writing–original draft, data curation, formal analysis, conceptualization, methodology. H.S. performed in data curation, formal analysis, methodology. S.W. performed in writing‐review and editing, project administration, conceptualization, supervision, resources, funding acquisition. Y.O. performed in data curation, methodology, investigation. X.Z. performed in methodology. B.H. performed in validation. X.Z. performed in validation. G.L. performed in methodology, resources. L.X. performed in project administration, conceptualization, writing‐review and editing. J.Z. performed in project administration, conceptualization, supervision, writing‐review and editing, funding acquisition.

## Supporting information



Supporting Information

## Data Availability

The data that support the findings of this study are available from the corresponding author upon reasonable request.
