# Corrigendum to “Hydrogel‐Transformable Probiotic Powder for Targeted Eradication of Helicobacter pylori with Enhanced Gastric Mucosal Repair and Microbiota Preservation”

**DOI:** 10.1002/advs.202517920

**Published:** 2025-10-28

**Authors:** Yongkang Lai, Hanchun Shen, Shige Wang, Yongliang Ouyang, Xinyuan Zhang, Bin Hu, Xiaoyi Zhang, Guisheng Li, Lizhi Xu, Jiulong Zhao

**Affiliations:** ^1^ Department of Gastroenterology Shanghai Institute of Pancreatic Diseases Changhai Hospital National Key Laboratory of Immunity and Inflammation Naval Medical University Shanghai 200433 P. R. China; ^2^ School of Materials and Chemistry University of Shanghai for Science and Technology Shanghai 200093 P. R. China; ^3^ Advanced Biomedical Instrumentation Centre Hong Kong Science Park Shatin New Territories Hong Kong SAR 999077 P. R. China; ^4^ Department of Mechanical Engineering The University of Hong Kong Hong Kong SAR 999077 P. R. China

Adv. Sci, 2025 Jun;12(23): e2500478.

https://doi.org/10.1002/advs.202500478.

In **Figure** **1**f–h, the exact positional correspondence between the first and second columns was unrecorded, making the red boxes in the first column unverifiable. These red boxes (erroneously copied during assembly and overlooked in revision) have been removed to avoid confusion. In Figure 1g, due to mislabeled files in bulk storage, the SEM image of *L. reuteri*@HTP was mistakenly used instead of HTP. In **Figure** **3**h, the Live/Dead staining image of *L. reuteri*@HTP powder (after 6 months) was incorrectly labeled “Before.” This has been corrected. These errors have now been corrected, and the revised figures are shown below.

**Figure 1:**
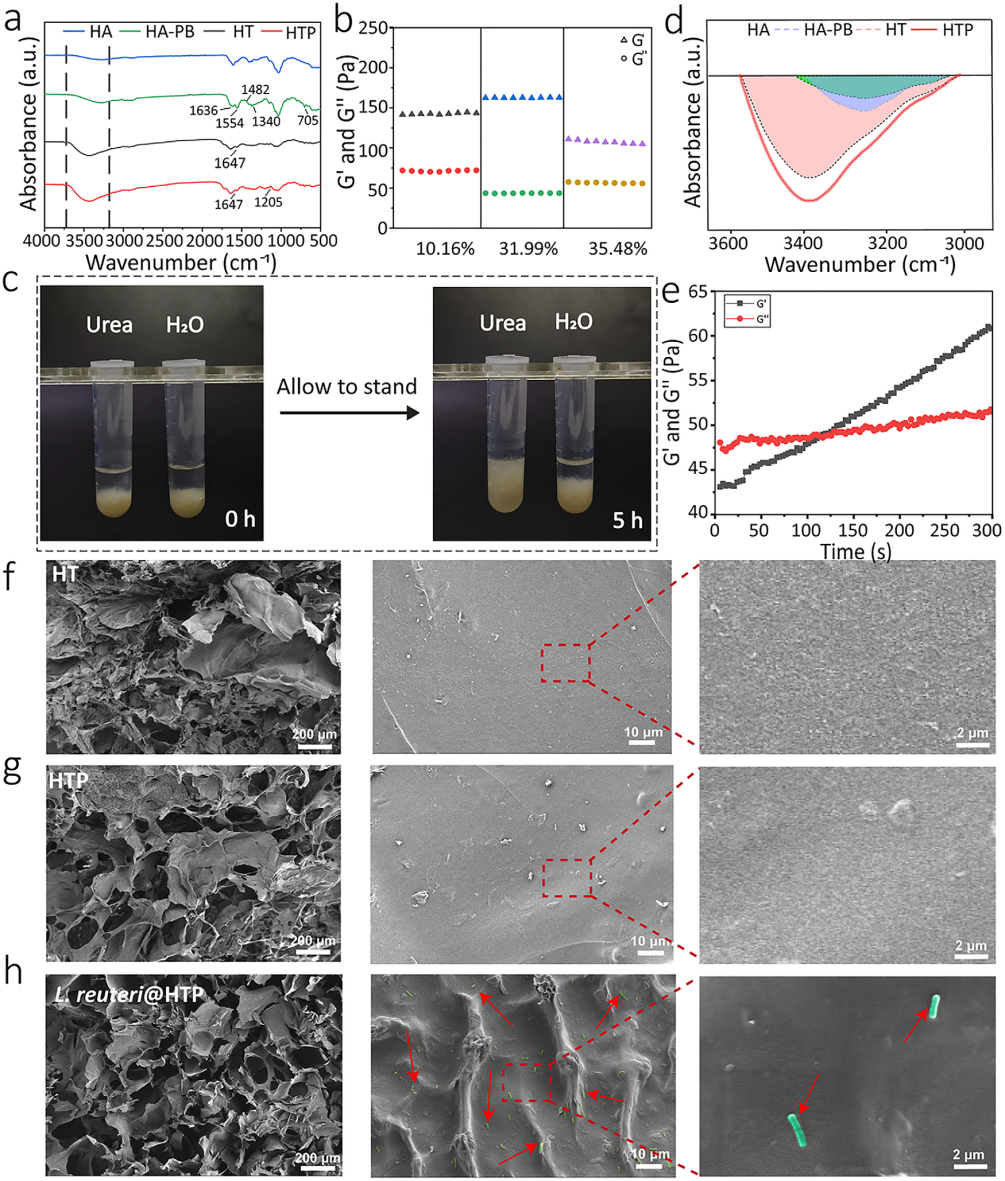
Synthesis and characterization of *L. reuteri*@HTP hydrogel. a) FTIR of HA, HA‐PB, HT hydrogel, and HTP hydrogel. b) Modulus of HT hydrogel. The HA‐PB grafting ratios are 10.16, 31.99, and 35.48%. c) Optical images of *L.reuteri*@HTP hydrogel in urea solution and water. d) FTIR peaks of hydrogen bonds of HA, HA‐PB, HT hydrogel, and HTP hydrogel. e) Dynamic time scanning rheological analysis of *L. reuteri*@HTP hydrogel. SEM images of the cross‐section of f) HT, g) HTP, and h) *L. reuteri*@HTP hydrogels (*L. reuteri* was marked in green using the Adobe Photoshop (2024 version) software and indicated using red arrows).

**Figure 3:**
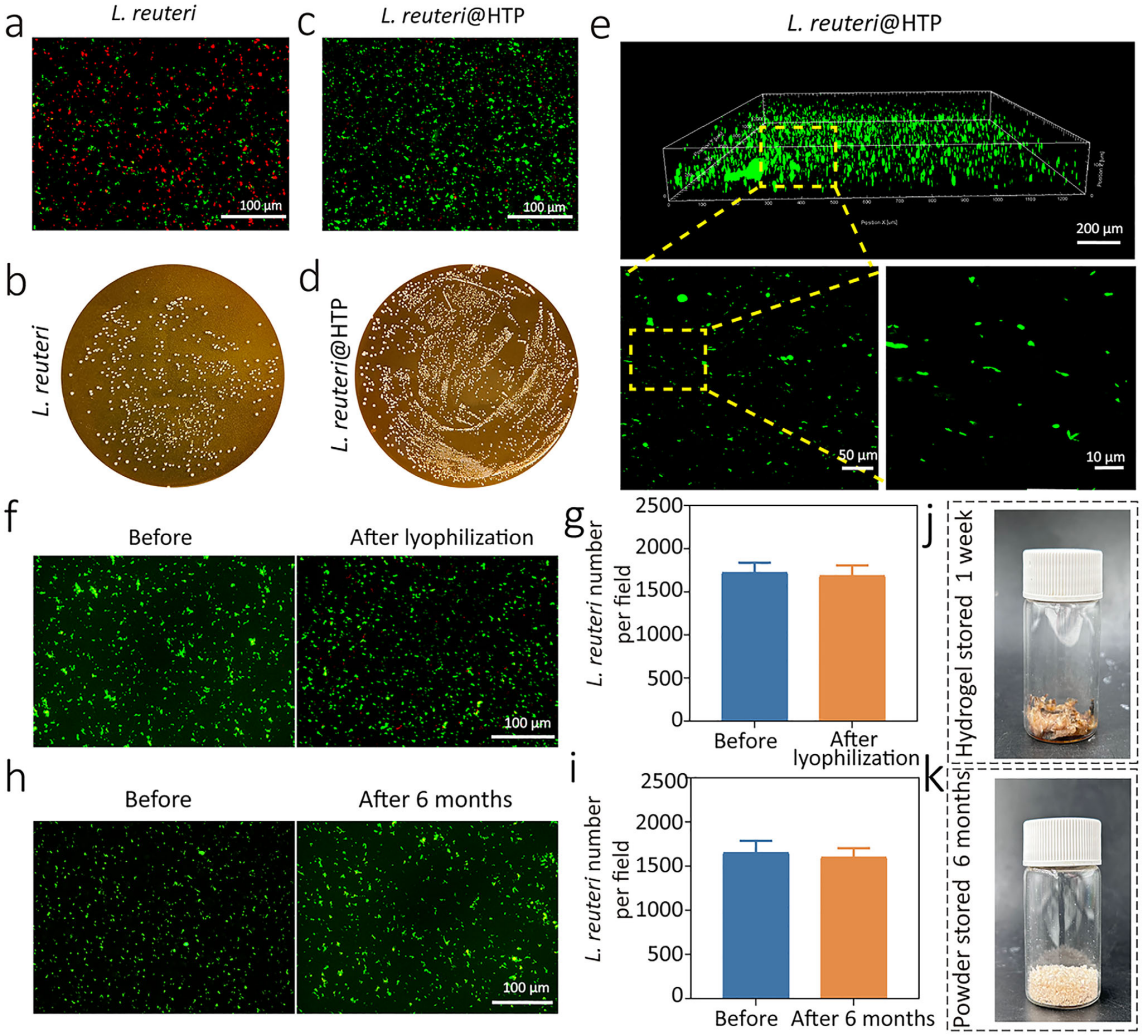
The ability of *L. reuteri*@HTP powder to maintain bacterial viability and extend storage time. a) Live/Dead bacterial staining and b) colony plate images of *L. reuteri* and transformed *L. reuteri*@HTP hydrogel after coculture with SGF + H_2_O_2_. c) Live/Dead bacterial staining and d) the quantitative analysis of the viable *L. reuteri* population in *L. reuteri*@HTP hydrogel and *L. reuteri*@HTP powder. e) Live/Dead bacterial staining of *L. reuteri* in pristine *L. reuteri*@HTP hydrogel and enlarged views. f) Live/Dead bacterial staining and g) the quantitative analysis of *L.reuteri* before (i.e., in *L. reuteri*@HTP hydrogel) and after lyophilization (i.e., in *L. reuteri*@HTP powder). h) Live/Dead bacterial staining and i) the quantitative analysis of the viable L. reuteri population in *L. reuteri*@HTP power before and after 6 months of storage. The appearance of j) *L. reuteri*@HTP hydrogel after 1 week of storage and k) *L. reuteri*@HTP powder after 6 months of storage. Data are presented as the mean ± SD (*n* = 3).

These changes do not affect the results or conclusions of this study. The authors would like to apologise for any inconvenience caused.

Y.L. and H.S.: contributed equally to this work.

