# Supplementary material for: Hydrogel‐Transformable Probiotic Powder for Targeted Eradication of Helicobacter pylori with Enhanced Gastric Mucosal Repair and Microbiota Preservation
Source: Adv Sci (Weinh). 2025 Mar 16;12(23):2500478. doi: 10.1002/advs.202500478 (PMC12199445; doi:10.1002/advs.202500478)
Supplement: Supplementary file 1 — Supporting Information [file ADVS-12-2500478-s001.docx]

## **Supplement materials**

**Hydrogel-Transformable Probiotic Powder for Targeted Eradication of *Helicobacter pylori* with Enhanced Gastric Mucosal Repair and Microbiota Preservation**

Yongkang Lai^1,†^, Hanchun Shen ^2,†^, Yongliang Ouyang ^2^, Xinyuan Zhang ^2^, Bin Hu ^2^, Xiaoyi Zhang,^3,4^, Shige Wang ^2,3,4,^*, Lizhi Xu ^3,4,^*, and Jiulong Zhao ^1,^*

1. Department of Gastroenterology, Shanghai Institute of Pancreatic Diseases, Changhai Hospital; National Key Laboratory of Immunity and Inflammation, Naval Medical University, Shanghai 200433, China
2. School of Materials and Chemistry, University of Shanghai for Science and Technology, Shanghai 200093, China
3. Advanced Biomedical Instrumentation Centre, Hong Kong Science Park, Shatin, New Territories, Hong Kong SAR 999077, P. R. China
4. Department of Mechanical Engineering, The University of Hong Kong, Hong Kong SAR 999077, P. R. China

^†^ These authors contributed equally to this paper. * Correspondence to: Prof. Wang, sgwang@usst.edu.cn; Prof. Xu, xulizhi@hku.hk; Prof Zhao, jlzhao9@163.com.

# Materials and Methods

## **1. Materials and characterizations**

Low molecular HA (molecular weight: 800 KDa-1.0 MDa, H293501), medium molecular HA (molecular weight: 1.0-1.8 MDa, H293485), and high molecular HA (molecular weight: >1.8 MDa, H293496) were purchased from Aladdin Reagent Co., Ltd. (Shanghai, China). TA (T25393) was purchased from Shanghai Yuanye Bio-Technology Co., Ltd. PB (A135767), 1,1-Diphenyl-2-picrylhydrazyl free radical (DPPH·) (D273092), 2-phenyl-4,4,5,5-tetramethylimidazoline-1-oxyl-3-oxide (PTIO·) (P160514), N-hydroxysuccinimide (NHS) (H109330), 1-(3-dimethylaminopropyl)-3-ethylcarbodiimide hydrochloride (EDC) (E106172), and PVA (M141491) were purchased from Aladdin Reagent Co., Ltd. (Shanghai, China). Amphotericin B (A8251), trimethoprim lactate (T9170), polymyxin B sulfate (P8350), vancomycin (V8050), and clarithromycin (C9490) were obtained from Sigma‒Aldrich (Shanghai, China). Simulated gastric fluid (SGF) (A7921) and simulated intestinal fluid (SIF) (A1791) were obtained from Solarbio Technology Co. (Beijing, China). Hieff UNICON® Universal Blue qPCR SYBR Green Master Mix (11184ES08), Cell Counting Kit-8 (CCK 8, CK04), and Live/Dead cell staining kit (40747ES76) were obtained from Yeasen Biotechnology (Shanghai, China) Co. Ltd. The urea test kit was obtained from Shandong Bomeda Biotechnology Co. The SYTO 9/PI Live/Dead bacterial staining kit (MX4234-40T) was obtained from Shanghai Moukang Biotechnology Co. (Shanghai, China). A field emission scanning electron microscope (ZEISS Sigma 300, Germany) was utilized for scanning electron microscopy (SEM) imaging. The chemical structure of the samples was analyzed using Fourier transform infrared (FTIR) spectroscopy with a Thermo Fisher Scientific Nicolet iS20 instrument (USA). ^1^H-NMR analysis was performed on an AVANCE III 400 MHz digital NMR nuclear magnetic resonance spectrometer (Bruker BioSpin, Karlsruhe, Germany) to analyze the structural features of the samples. Gastric mucosal epithelial cells (HEF-145) and the *H. pylori* strain CagA(+) ATCC43504 and PMSS1 were obtained from the First Affiliated Hospital of Nanchang University. RAW264.7 cells were purchased from the Cell Bank of the Chinese Academy of Sciences (Beijing, China). *L. reuteri* strains were obtained from Zhenjiang Tianyi Biotechnology Co. (Jiangsu, China) and grown in Lactobacillus MRS media at 37°C. All animal procedures in this study were approved by the Ethics Committee of the First Affiliated Hospital of Naval Medical University (Shanghai, China, Approval Number: CHFC(A.E)2023-017). C57BL/6 male mice (5–6 weeks old, weighing 15–25 g) were obtained from Jiangsu GemPharmatech LLC and maintained in a specific pathogen-free (SPF) environment.

## **2. Preparation of hydrogel and hydrogel powder**

**Preparation of HA modified with PB (HA-PB):** 1 g of medium molecular HA was fully dissolved in 100 mL of deionized water. Then, 0.86 g of PB, 0.58 g of NHS, and 0.96 g of EDC were added to the HA solution to obtain a yellow viscous solution by stirring for 48 h at room temperature. Finally, the solution was placed in a dialysis bag with a cutoff molecular weight of 3000 Da and dialyzed in deionized water for 5 d to remove unreacted impurities. At the end of dialysis, the product was lyophilized to obtain HA-PB. To prepare HA-PB with lower and higher grafting ratios, the concentrations of PB and EDC/NHS were halved and increased to 4/3 of the original, respectively. The PB grafting ratio was calculated by integrating the peak areas of the ^1^H-NMR spectra as follows (I_a_ and I_b_ are the areas of peak a and peak b respectively):

PB grafting ratio (%) = I_a_/I_b_ × 100%.

**Preparation of *L. reuteri*@PVA:** PVA was dissolved in deionized water to prepare the PVA solution (2 mg/mL). Then, 2 mL of *L. reuteri* bacterial suspension (1 × 10^8^ CFU/mL, total 4 mg of *L. reuteri*) was mixed with 2 mL of PVA solution and stirred thoroughly for 1 min to ensure the uniform encapsulation of the bacteria within the PVA. The mixture was then lyophilized to obtain *L. reuteri*@PVA powder.

**Preparation of hydrogels:** HA-PB solution (3 mL, 4 mg/mL) was mixed with TA solutions (1 mL, 4 mg/mL) to get the HT hydrogel precursor solution. To prepare HTP hydrogel, TA solution (1 mL, 4 mg/mL), HA-PB solution (3 mL, 4 mg/mL), and PVA solution (1 mL, 2 mg/mL) were mixed to get the HTP hydrogel precursor solution. To prepare the pristine *L. reuteri*@HTP hydrogel, HA-PB solution (3 mL, 4 mg/mL) and TA solution (1 mL, 4 mg/mL) were mixed with *L. reuteri*@PVA solution (1 mL, 2 mg/mL, containing 1 mg *L. reuteri*) to get the *L. reuteri*@HTP hydrogel precursor solution. The above precursor solutions were stirred for 1 min to obtain the corresponding hydrogel. To verify the existence of hydrogen bonds in the *L.reuteri*@HTP hydrogel, we soaked the synthesized *L.reuteri*@HTP hydrogel in urea (6 mol/L) and double distilled water, respectively. Images of the *L.reuteri*@HTP hydrogel were captured before and after 5 h of immersion.

**Preparation of HTP powder and *L. reuteri*@HTP powder:** To prepare HTP powder and *L. reuteri*@HTP powder, the pristine HTP and *L. reuteri*@HTP hydrogels were frozen overnight at -20°C and lyophilized in a standard lyophilizer (SCIENTZ-12N, Ningbo Scientz Biotechnology Co., LTD, Ningbo, China). The lyophilized hydrogels were ground into the powders using a grinder (LG-30 g, Ruian Baixin Pharmaceutical Machinery Co., LTD, Ruian, China). 5 mL of *L. reuteri*@HTP hydrogel precursor solution was lyophilized and ground into powder to yield about 2 mg of *L. reuteri*@HTP powder, of which the mass of *L. reuteri* was about 1 mg, and the masses of HA-PB, TA, and PVA were about 0.67 mg, 0.22 mg, and 0.11 mg, respectively.

## **3. The swelling ability of the hydrogel**

To characterize the swelling of the hydrogel, the lyophilized hydrogel was immersed in 5 mL of water and placed in a simulated physiological environment at 37°C with oscillation (100 rpm). At predetermined time points, the hydrogel was removed, excess surface water was blotted with filter paper, and the swelling ratio of the hydrogel was calculated using the following formula:

Swelling ratio (%) = (W_a_ -W_b_)/W_a_×100%

Where W_a_ represents the mass of the hydrogel after swelling, and W_b_ represents the initial mass of the hydrogel.

## **4. Porosity of hydrogel**

The porosity of the hydrogel was calculated using the ethanol displacement method. First, the mass and volume of the lyophilized hydrogel were recorded, followed by immersion in anhydrous ethanol. After 2 h, the surface ethanol was removed with filter paper, and the hydrogel was weighed. The porosity was calculated using the following formula:

Porosity = (W_e_ - W_d_)/(V × ρ_e_) × 100%

Where W_e_ is the mass of the ethanol-saturated hydrogel, W_d_ is the mass of the dry hydrogel, V is the volume of the hydrogel, and ρ_e_ is the density of ethanol.

## **5. Degradation studies of hydrogel**

The HT, HTP, and *L. reuteri*@HTP powder (0.3 g) was mixed with water (0.9 mL) to obtain the transformed HT, HTP, and *L. reuteri*@HTP hydrogels, respectively. Then, 0.15 g of the transformed hydrogel was immersed in 10 mL of SGF, SGF containing 0.1% H_2_O_2_ (w/v, H_2_O_2_ + SGF, to mimic gastritis microenvironment) and SIF, respectively. The above-mixed solutions were then incubated at 37°C with oscillation (100 rpm). These samples were photographed after 0 min, 30 min, 60 min, 180 min, and 720 min oscillation, and weighed after 2 h, 4 h, 6 h, 8 h, 12 h, and 24 h. The *in vitro* degradation ratio was calculated by dividing the mass of the degraded hydrogel by its original mass.

## **6. Rheological testing of hydrogel**

Dynamic time scanning rheological analysis was examined with a DHR-2 rheometer (HAAKE MARS III, Germany). Briefly, 500 μL of the tested hydrogel precursor solution was dispensed onto the mold of the rotational rheometer (P20 TiL, diameter 20 mm). Then, the machine was started and tested with a plate spacing of 1 mm between the molds, a temperature of 37°C, a frequency of 1 Hz, and a time-modulus test mode. The dynamic energy storage modulus (G′) and loss modulus (G′′) of the samples at 1% strain were recorded in the frequency scanning test.

## **7. Cells, bacterial strains, and mice**

HEF-145 and Raw264.7 were cultured in DMEM/F12 supplemented with 10% FBS and 1% penicillin/streptomycin under standard conditions (5% CO_2_, 37°C). Two H. pylori strains were utilized: CagA (+) ATCC 43504 for in vitro studies and PMSS1 for *in vivo* experiments. Both were cultured on blood agar plates supplemented with 5% sheep blood and a mixed antibiotic solution (5 µg/mL trimethoprim lactate, 5 µg/mL amphotericin B, 5 µg/mL polymyxin B sulfate and 10 µg/mL vancomycin) under microaerobic conditions (10% CO₂, 5% O₂) at 37°C. After two passages, they were prepared for experiments using liquid culture in Brucella broth supplemented with 10% FBS and 0.5% antibiotics, incubated on a micro shaker (100 rpm). The animal experiments were conducted with approval from the Ethics Committee of the First Affiliated Hospital of Naval Medical University (Approval No. CHFC (A.E.) 2023-017). To establish an *H. pylori* infection model, mice were orally administered an *H. pylori* (PMSS1) suspension (1 × 10⁸ CFU/mL, 300 μL) every other day for two weeks. Five mice were randomly selected and euthanized after four weeks of normal feeding for gastric tissue collection. Gastric tissue plating, H&E staining, and the rapid urease test were used to confirm the successful establishment of the *H. pylori* infection model.

## **8. Viability testing of *L. reuteri***

*L. reuteri* and *L. reuteri*@HTP hydrogel were placed in SGF containing 0.1% H_2_O_2_ (w/v, H_2_O_2_ + SGF) solution and incubated at 37°C with oscillation (100 rpm) for 2 h. The precipitates were centrifuged (1500 rpm), and the surviving *L. reuteri* was evaluated by Live/Dead bacterial staining and plate coating. The observation was performed using an ortho-fluorescence microscope (MshOt, MF43-N, China). Further, to test the viability of *L. reuteri* in *L. reuteri*@HTP hydrogel, we labeled *L. reuteri* with SYTO 9 according to the instructions and prepared the SYTO 9-labeled *L. reuteri*@HTP hydrogel. After 24 h of resting under the standard condition, the confocal laser scanning microscopy (CLSM, ZEISS LSM880, Germany) was used to assess the fluorescence of *L. reuteri* inside the *L. reuteri*@HTP hydrogel. Finally, pristine *L. reuteri*@HTP hydrogel and *L. reuteri*@HTP powder were separately placed in a glass bottle. These two bottles were kept their lids open and stored at a 4 ℃ refrigerator for up to 6 months, during which their appearance was observed and recorded using a photographer. After 6 months, the viability of *L. reuteri* was reassessed by Live/Dead bacterial staining.

## **9. Cell compatibility and Live/Dead Cell Staining assay**

Gastric epithelial (HEF-145) cells were seeded into 96-well plates, with each well containing 1 × 10^4^ cells. After overnight adherence, the culture medium was replaced with 100 μL of medium containing different concentrations of *L.reuteri*@HTP hydrogel. Subsequently, the cells were cultured for 24 h, 48 h, or 72 h. Cell viability was determined using the CCK-8 assay, where 100 μL of 10% CCK-8 dye solution was added to each well. Following a 1h incubation at 37°C, the absorbance of the water-soluble formazan dye was measured at 450 nm using a microplate reader (Molecular Devices SpectraMax® i3, USA). Each experimental group included three replicate wells and cell viability was calculated using the following formula:

Cell viability (%) = (OD_experimental groups_ - OD_blank groups_)/(OD_control groups_ - OD_blank groups_) × 100%.

For the Live/Dead Cell Staining assay, calcein-AM, and PI dyes were added to the culture medium after coculturing the materials with cells. After a 30-min incubation at 37°C, the cells were observed under a fluorescence-inverted microscope (Leica, DMIL LED, Germany).

## **10. Apoptosis analysis by flow cytometry**

HEF-145 cells were seeded into six-well plates at a density of 1 × 10^6^ cells per well. After overnight adherence, the medium was replaced with a culture medium containing different concentrations of *L.reuteri*@HTP. Following a 24-hour treatment, the cells were collected and washed twice with PBS. Subsequently, the cell pellet was resuspended in the sample solution and incubated with 5 µL of Annexin V-FITC (Yeasen, 40302ES50, China) in the dark for 15 min, followed by an additional 5-min incubation with 10 µL of propidium iodide (PI, Yeasen, 40302ES50, China). Apoptotic cells were then detected using flow cytometry (Agilent, NovoCyte 3110, USA).

## **11. Hemolysis assay**

Fresh rat blood was obtained and centrifuged at 5000 rpm for 5 min to isolate the cellular components. The resulting pellet was resuspended, and 2 mL of blood was diluted to a final volume of 50 mL using physiological saline. For control groups, 300 μL of this diluted blood was combined with either 1.2 mL of physiological saline for the negative control or an equal volume of deionized water for the positive control. The experimental groups were prepared by adding 300 μL of red blood cells to 1.2 mL of *L.reuteri*@HTP hydrogel solution at various concentrations (5 mg/mL, 10 mg/mL, 15 mg/mL, 20 mg/mL). After a 2 h incubation at 37°C, the absorbance of the supernatant was quantified at 540 nm with a microplate reader. The hemolysis rate was then determined employing the following formula:

Hemolysis ratio (%) = (OD_experimental group_ - OD_negative control group_)/(OD_positive control group_ - OD_positive control group_) × 100%.

## **12. *In vitro* adhesion study of *L. reuteri* to *H. pylori***

*L. reuteri* (5 mL, 1 × 10^8^ CFU/mL) and *H. pylori* (ATCC43504, 5 mL, 1 × 10^8^ CFU/mL) were mixed thoroughly. Subsequently, 1 mL of the mixed suspensions were placed into 9 mL of PBS and 9 mL of SGF containing 0.1% H_2_O_2_ (w/v, SGF + H_2_O_2_) solution, respectively. After shaking (100 rpm) at room temperature for 20 min, the suspensions were centrifuged (1500 rpm, 5 min) to remove the supernatant, and the precipitate was fixed in 4% glutaraldehyde and dehydrated in a graded ethanol series, followed by gold sputtering for observation of bacterial morphology under SEM. The bacteria were then stained using Adobe Photoshop (2024 version) according to their shape.

## **13. *In vitro* anti-*H. pylori* study of *L. reuteri*@HTP** **powder**

The *in vitro* anti-*H. pylori* ability was evaluated through plate coating and Live/Dead bacterial staining. *H. pylori* (ATCC43504, 1 mL, 1 × 10^7^ CFU/mL) were cocultured with PBS (100 μL, control), HTP powder (100 mg), *L. reuteri* (100 mg), and *L. reuteri*@HTP powder (200 mg) in a system consisting of 10 mL of Brucella broth, 1 mL of FBS, and 50 μL of mixed antibiotics. The coculture was oscillated and incubated for 24 h. Subsequently, the coculture liquid was diluted, and 50 μL of it was evenly spread on blood agar plates. The plates were then incubated for 72 h, after which the colonies on the plates were photographed and counted. To further study the anti-*H. pylori* efficiency*,* 50 μL of the diluted coculture liquid was centrifuged and stained with the SYTO 9/PI Live/Dead bacterial staining reagent according to the instructions. Finally, the stained bacteria were recorded using a fluorescence microscope (MshOt, MF43-N, China). Then, ten randomly selected visual fields from each group were examined to quantify bacterial counts.

## **14. *In vitro* free radical-scavenging assays**

The capacity of the *L. reuteri*@HTP powder to scavenge reactive nitrogen species (RNS) and reactive oxygen species (ROS) was evaluated by assessing their efficacy against nitrogen-centered radicals (DPPH·) and oxygen-centered radicals (PTIO·), respectively. For the DPPH· assay, varying qualities (10 mg, 20 mg, 30 mg, and 40 mg) of *L. reuteri*@HTP powder were introduced to 2 mL of DPPH· solution. In the PTIO· assay, different qualities (10 mg, 20 mg, 30 mg, and 40 mg) of *L. reuteri*@HTP powder were mixed with 2 mL of PTIO· solution. The above mixtures were incubated at 37°C for 30 min in a light-protected environment using a UV spectrophotometer. The free radical scavenging ratio was calculated by employing the formula:

Scavenging ratio = (A_0_ - A_b_)/A_0_ × 100%.

In this equation, A_0_ is the absorbance of the blank (DPPH·+ ethanol or PTIO· + ethanol) and A_b_ is the absorbance of the experimental group (DPPH· + ethanol + *L. reuteri*@HTP powder or PTIO· + ethanol + *L. reuteri*@HTP powder).

## **15. Intracellular free radical-scavenging study**

RAW264.7 cells were inoculated in 6-well culture plates at a density of 5 × 10^5^ cells per well. After 24 h of incubation, the cell culture medium was replaced by fresh medium (control group), fresh medium containing H_2_O_2_ (1 mM), *L. reuteri* (10 mg/mL) + H_2_O_2_ group (1 mM), or *L. reuteri*@HTP powder (20 mg/mL)) + H_2_O_2_ group (1 mM), and the cells were cultured for another 6 h. Finally, the cells were incubated with the ROS-sensitive fluorescent dye 2',7'-dichlorofluorescin diacetate (DCFH-DA) (Beyotime Biotechnology, China) for 20 min. Subsequently, the cells were washed three times with PBS by centrifugation. Intracellular ROS levels were observed using an inverted fluorescence microscope (Leica, DMIL LED, Germany), and flow cytometry analysis (Agilent, NovoCyte 3110, USA) was performed to quantify intracellular ROS levels. The excitation wavelength was set at 488 nm, and the emission wavelength was set at 525 nm.

## **16. Cell migration assay**

HFE-145 were evenly seeded onto a 6-well plate and allowed to grow until they reached confluence. A 10 µL pipette tip was used to create scratch wounds on the cell monolayer, followed by three gentle PBS washes to eliminate detached cells. The cells were then incubated at 37°C with serum-free medium supplemented with either HTP (10 mg/mL) or *L. reuteri*@HTP powder (20 mg/mL). Images were taken at 0, 12, 24, and 36 h post-incubation. The cell migration rate was calculated as the percentage reduction in the scratch wound area from the original area to the area at the time of testing.

## **17. *In vivo* adhesion evaluation of *L. reuteri* to *H. pylori***

*L. reuteri* was labeled with Cy7 (MCE, HY-D0825) according to the instructions and then the Cy7-labeled *L. reuteri*@HTP powder was prepared using the same method as *L. reuteri*@HTP powder and stored in the dark. Subsequently, fasted normal and *H. pylori*-infected mice (n = 3) were administered Cy7-labeled *L. reuteri*@HTP powder (20 mg) via gavage. Eight hours after gavage, the mice were euthanized, and the gastric tissues were harvested for imaging using an IVIS imaging system (Tanon ABL X6, China). Note that Cy7 was used for the IVIS imaging system.

## **18. *In vivo* anti-*H. pylori* evaluation**

The mice infected with *H. pylori* were randomly divided into 5 groups (n = 6): PBS (0.5 mL), HTP powder (0.5 g/kg/day), *L. reuteri* (0.5 g/kg/day), antibiotics (clarithromycin, 14.3 mg/kg/day), and *L. reuteri*@HTP powder (1 g/kg/day). PBS, HTP powder, *L. reuteri*, antibiotics, and *L. reuteri*@HTP powder were administered orally once daily for one week. Another 6 healthy mice were assigned as the 6^th^ group (control group) for comparison. Finally, all mice were euthanized, and their stomach tissues were collected. One section of the stomach was homogenized in sterile distilled water, and the resulting homogenate was used in a plate coating assay to evaluate *H. pylori* activity *in vivo*. The remaining stomach tissue was processed for H&E staining, and the *H. pylori* content in the gastric tissue was observed microscopically using an MshOt MF43-N microscope (China).

## **19. *In vivo* biocompatibility assay of *L. reuteri*@HTP** **powder**

After the *in vivo* anti-*H. pylori* evaluation (section 18), the heart, liver, spleen, lung, and kidney of the euthanized mice were collected for H&E staining to assess animal safety. Blood samples were collected from the eyes of the mice for routine blood tests, and serum was obtained for liver and kidney function tests to evaluate changes in blood and liver/kidney function parameters.

## **20. *In vivo* mucosal repair assessment**

Normal mice and infected mice after the *in vivo* anti-*H. pylori* evaluation (section 18) were euthanized, and gastric tissues were extracted. A portion of the gastric tissue was subjected to immunofluorescence staining to assess the status of gastric inflammation repair *in vivo* and observed using an ortho-fluorescence microscope (MshOt, MF43-N, China). β-catenin and proliferation cell nuclear antigen (PCNA) staining were conducted to evaluate the apoptosis of HEF-145 cells, while Occludin-1 and Claudin staining were performed to assess gastric mucosal repair. Another portion of the gastric tissue was subjected to RNA extraction according to the according to the TRIzolTM protocol, adhering strictly to the manufacturer's instructions, and the relative expression levels of β-catenin, Ki-67, Occludin-1, and Claudin were detected via qPCR.

## **21. *In vivo* anti-inflammatory tests**

The *in vivo* anti-inflammatory effects of *L. reuteri*@HTP powder were assessed using enzyme-linked immunosorbent assay (ELISA) and H&E staining. In brief, serum samples from treated mice (section 18) were diluted according to the instructions of the ELISA kit (FAM-INF-1, RayBiotech, Inc., Guangzhou, China) and incubated with ELISA detection solutions (MPO activity, IL-18, IL-17, IL-12, IL-10, IL-4, IL-1β, IL-2, and TGF-α). The absorbance was measured at 450 nm using a microplate reader (Molecular Devices SpectraMax® i3, USA). For H&E staining, gastric tissues from treated mice were subjected to H&E staining, and the degree of inflammation in H&E images was evaluated according to the updated Sydney system.

## **22. Gut microbiota analysis**

On the morning following the final treatment, the mice after the *in vivo* anti-*H. pylori* evaluation (section 18) were placed in a clean, sterile container to collect fresh fecal samples, and the number and diversity of the bacteria were determined by 16S rRNA sequencing. The analysis was conducted by Shanghai Oebiotech Biotechnology Co. (Shanghai, China). Briefly, genomic DNA was extracted from samples using the Magan Pure Soil DNA LQ Kit, following the manufacturer's protocol. DNA concentration and purity were assessed via NanoDrop 2000 spectrophotometer and agarose gel electrophoresis, with samples stored at -20°C. For bacterial 16S rRNA gene amplification, the V3-V4 region was targeted using barcoded primers (343F and 798R) and Takara Ex Taq polymerase. Amplicons were purified, quantified using Qubit, and libraries were prepared with the NEBNext® Ultra™ II FS DNA Library Prep Kit for sequencing on the Illumina NovaSeq6000 platform (250 bp paired-end reads). Post-sequencing, primers were trimmed using Cutadapt, and quality control, denoising, and chimera removal were performed with QIIME 2’s DADA2 plugin, generating amplicon sequence variant (ASV) tables. Taxonomic annotation employed the Silva v138 database via QIIME 2’s q2-feature-classifier plugin. Diversity metrics (α and β) and differential abundance analyses (LEfSe) were computed using QIIME 2.

## **23. Statistical analysis**

All the data are presented as the mean ± standard deviation (SD). Statistical analysis was performed using IBM SPSS Statistics 25.0 (IBM Corp, Chicago, IL, USA). Graphs were generated using GraphPad Prism 9.5 (GraphPad Software, La Jolla, CA, USA) and OriginLab 9.0 (OriginLab Corporation, Northampton, MA, USA). Differences between two groups were compared using Student's t-test, while differences among three or more groups were compared using one-way analysis of variance (ANOVA). A *p-value* < 0.05 was considered to indicate statistical significance (ns: not significant, **p <* 0.05, ***p <* 0.01, and ****p <* 0.001).

**Supplement Figure**


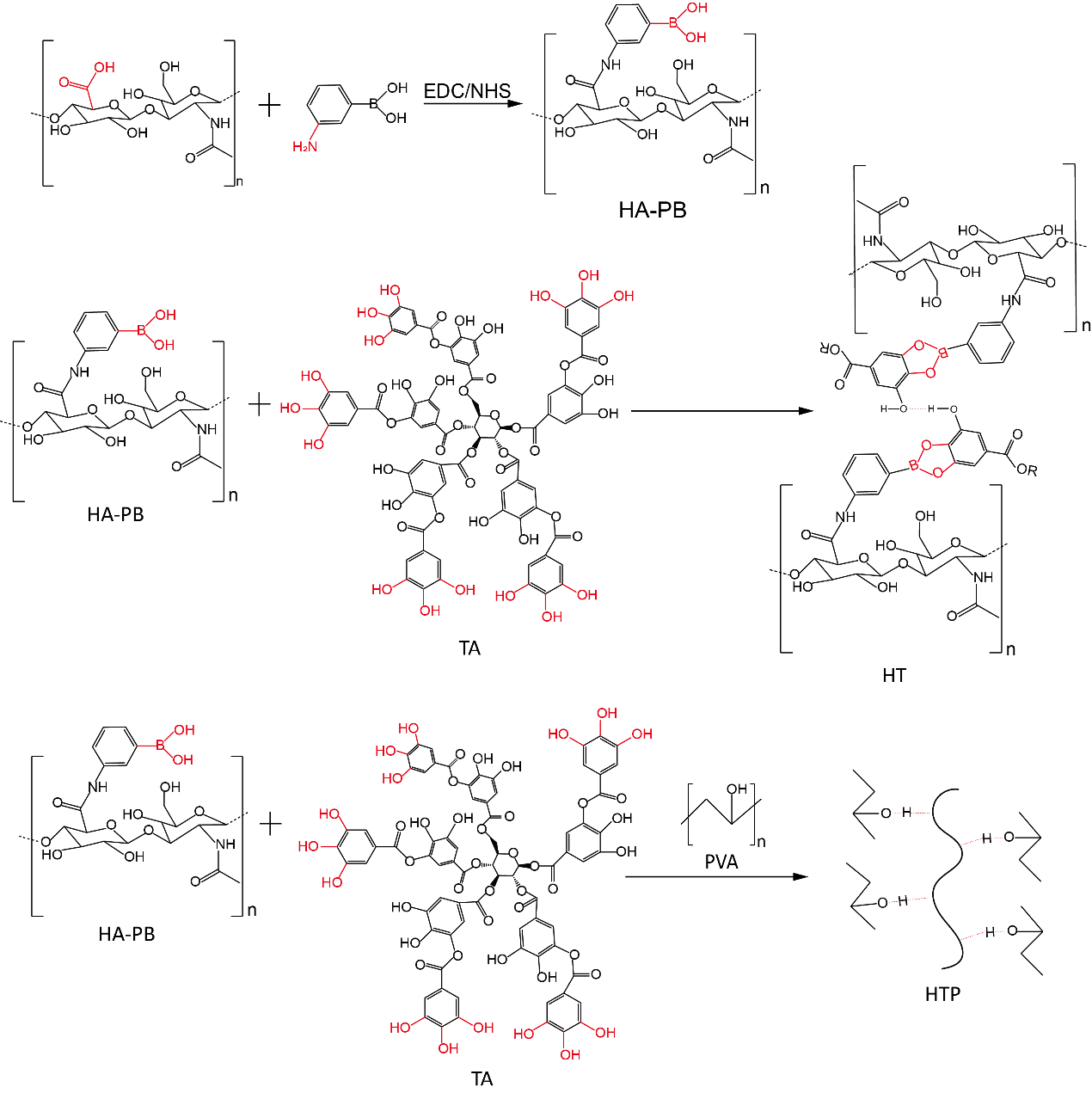


**Figure S1**. The synthesis procedures of sodium hyaluronate (HA)-3-aminophenylboronic acid (PB), HT hydrogel, and HTP hydrogel.


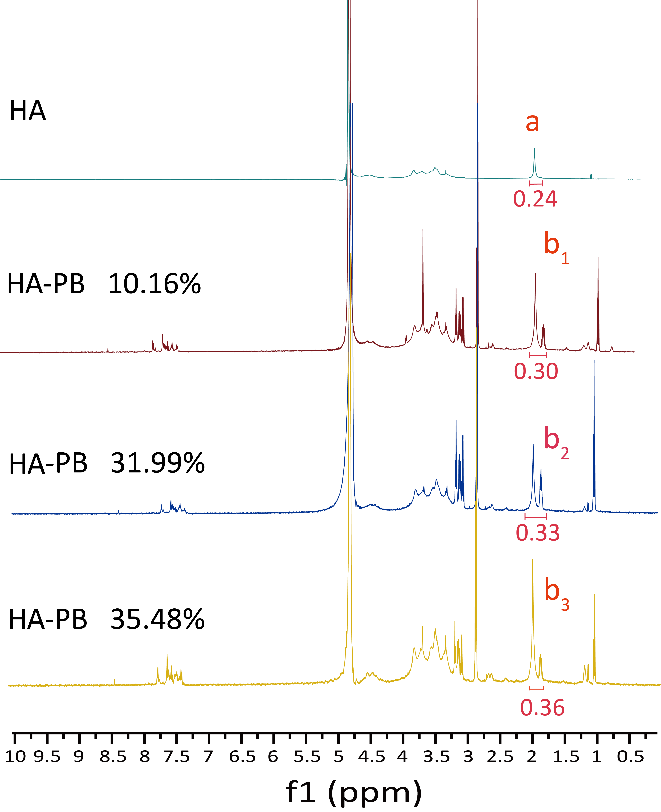


**Figure S2**. ^1^H nuclear magnetic spectrum (^1^H NMR) of HA and HA-PB with PB grafting ratios of 10.16%, 31.99%, and 35.48%.

Synthetic procedures: (a) 1 g of HA was dissolved fully in 100 mL of deionized water, then, 0.43 g of PB, 0.29 g of NHS, and 0.48 g of EDC were added; (c) 1 g of HA was dissolved fully in 100 mL of deionized water, then, 1.14 g of PB, 0.77 g of NHS, and 1.28 g of EDC were added. After stirring the mixture at room temperature for 24 h, a yellow viscous solution was obtained. This solution was placed into a dialysis bag with a molecular weight cutoff of 3000 Da and dialyzed against deionized water for 5 days to remove unreacted PB and EDC/NHS.


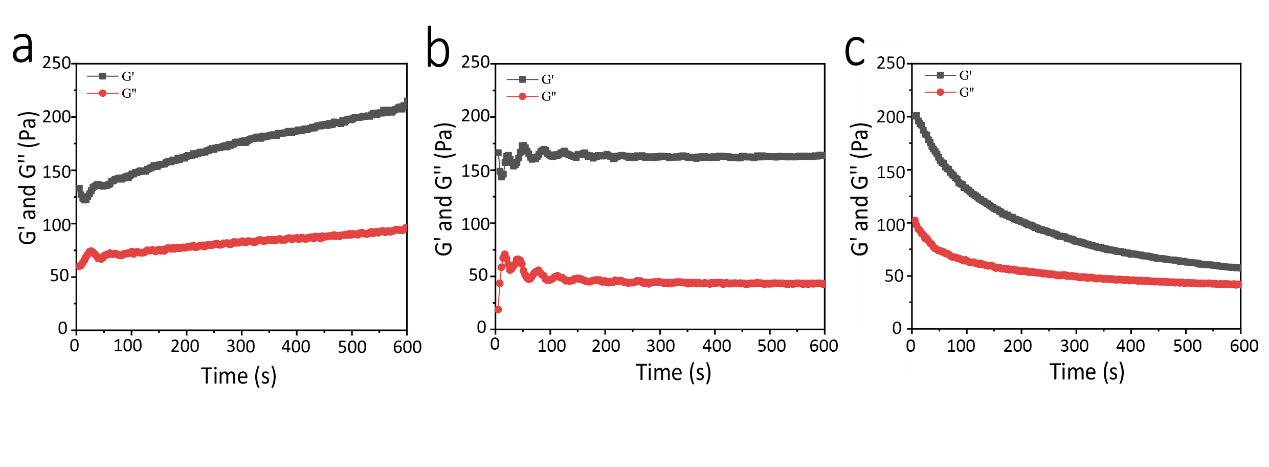
**Figure S3.** Dynamic time-scan rheology of HT hydrogels. (a) HA-PB with a PB grafting ratio of 10.16%; (b) HA-PB with a PB grafting ratio of 31.99%; (c) HA-PB with a PB grafting ratio of 35.48%.


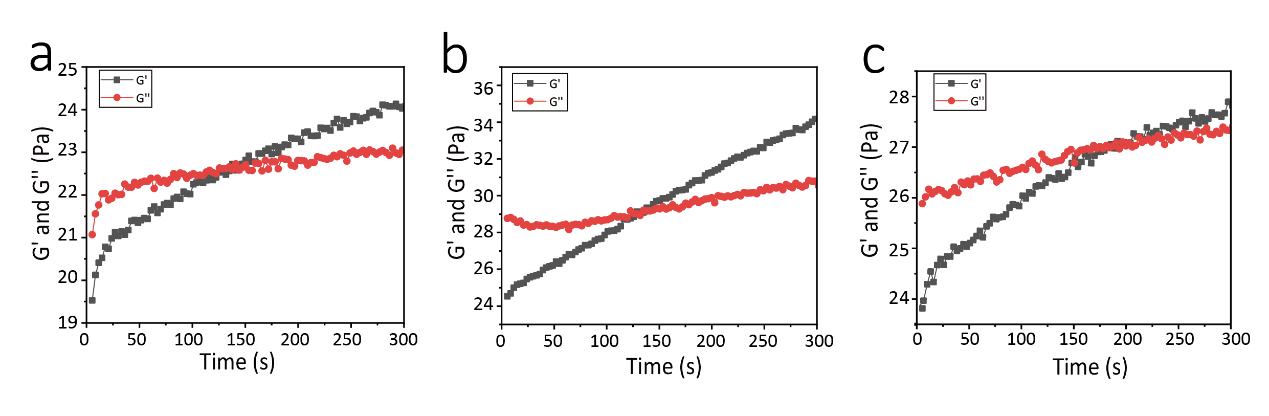


**Figure S4**. Dynamic time-scan rheology of HT hydrogels. To prepare HT hydrogels, 600 μL of HA-PB solution (4 mg/mL) was mixed with 200 μL of TA solution (4 mg/mL), where the molecular weight of HA is (a)＜1.0 MDa (low molecular weight), (b) between 1.0-1.8 MDa (medium molecular weight) and (c) ＞1.0 MDa (high molecular weight).


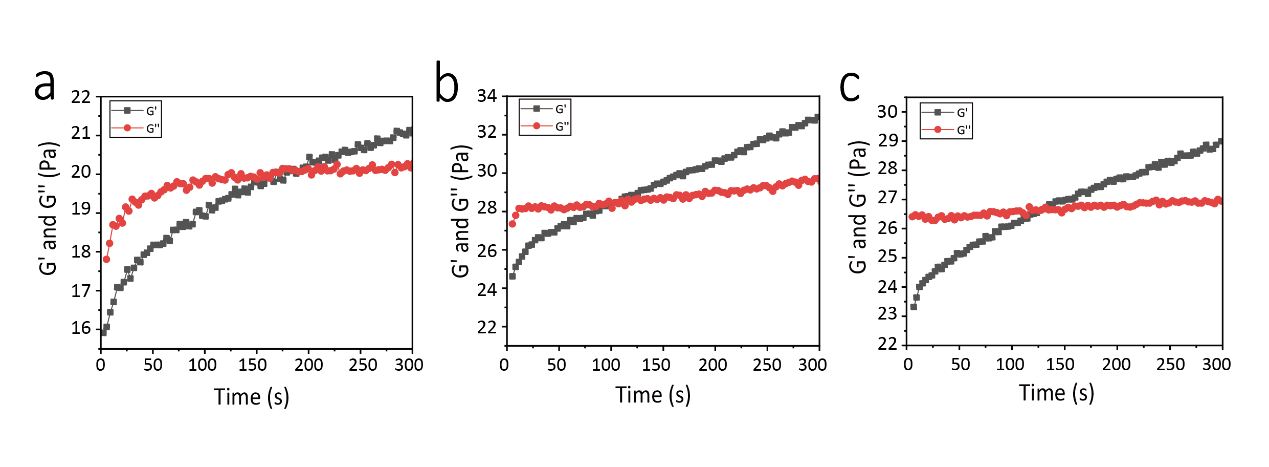


**Figure S5.** Dynamic time-scan rheology of HT hydrogels. To prepare HT hydrogels, 600 μL of HA-PB solution (4 mg/mL) was mixed with (a) 100 μL, (b) 200 μL, and (c) 300 μL of TA solution (4 mg/mL).


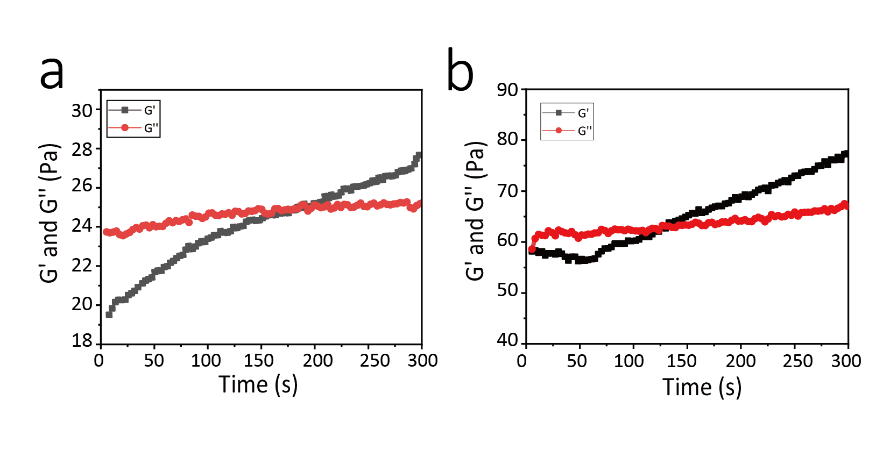


**Figure S6**. Dynamic time-scan rheology of HTP hydrogels. To prepare HTP hydrogels, HA-PB solution (600 μL, 4 mg/mL), TA solution (200 μL, 4 mg/mL), and PVA solution (2 mg/mL, (a) 100 μL and (b) 300 μL) were mixed and stirred for 1 min.


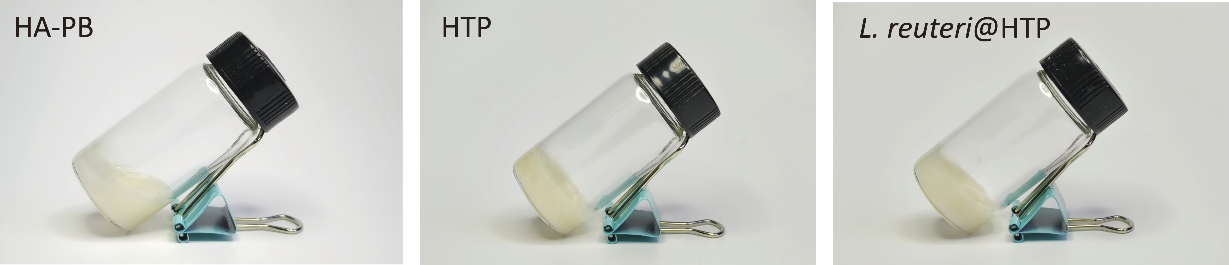


**Figure S7**. Optical images of HA-PB solution, HTP hydrogel, and *L. reuteri*@HTP hydrogel.


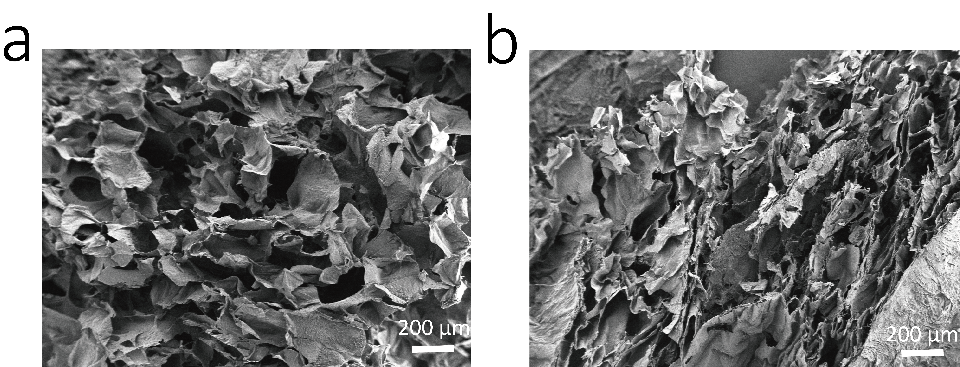


**Figure S8.** SEM of different HTP hydrogels with porosities of 47.45% (panel a) and 33.67% (panel b), corresponds to Figure S6a and Figure S6b, respectively.


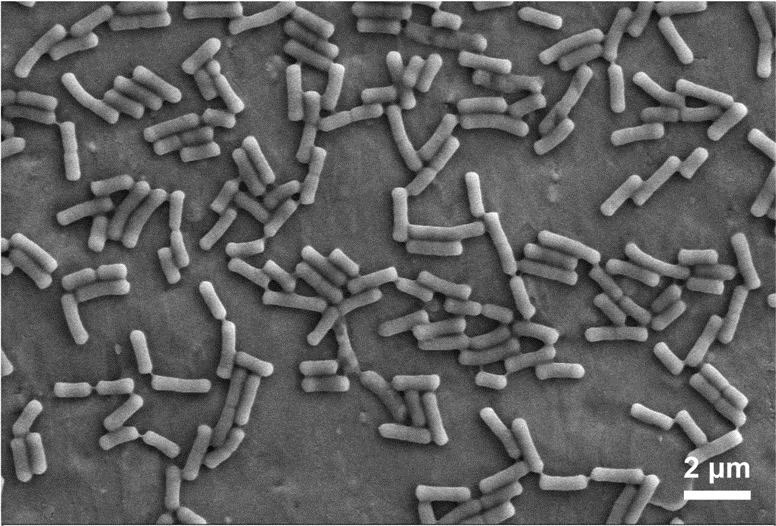


**Figure S9**. SEM image of *L. reuteri*.


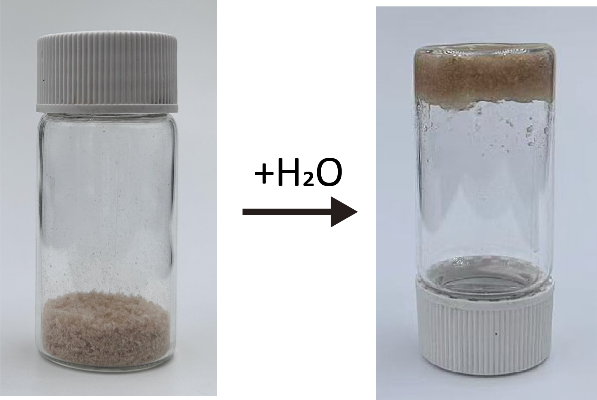


**Figure S10**. The picture of the HTP powder-hydrogel transformation process.





**Figure S11**. Dynamic time-scan rheology of the transformed hydrogel.


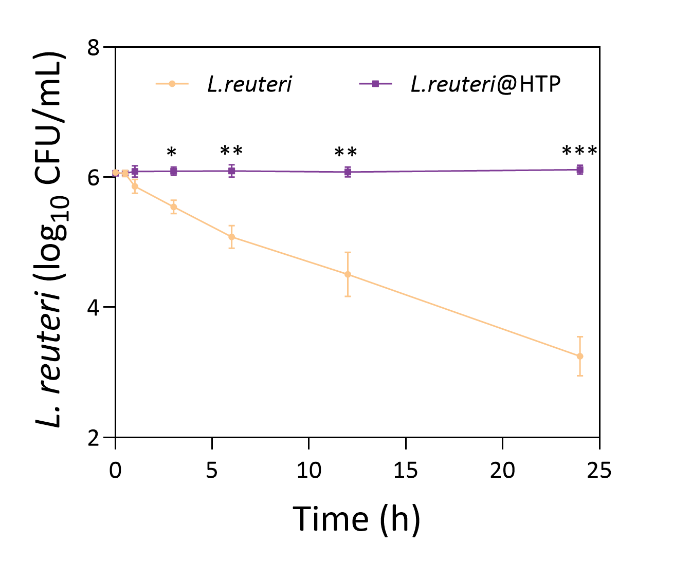


**Supplement Figure 12**. *L. reuteri*@HTP containing *L. reuteri* at a concentration of 10⁶ CFU/mL and free *L. reuteri* (10⁶ CFU/mL) were separately placed in a simulated gastritis environment. At 0 min, 30 min, 1 h, 6 h, 12 h, and 24 h, the supernatant was removed by centrifugation, and the precipitate was resuspended and plated. Bacterial viability in each group was then assessed by colony counting. **p* < 0.05, ***p* < 0.01, and ****p* < 0.001.


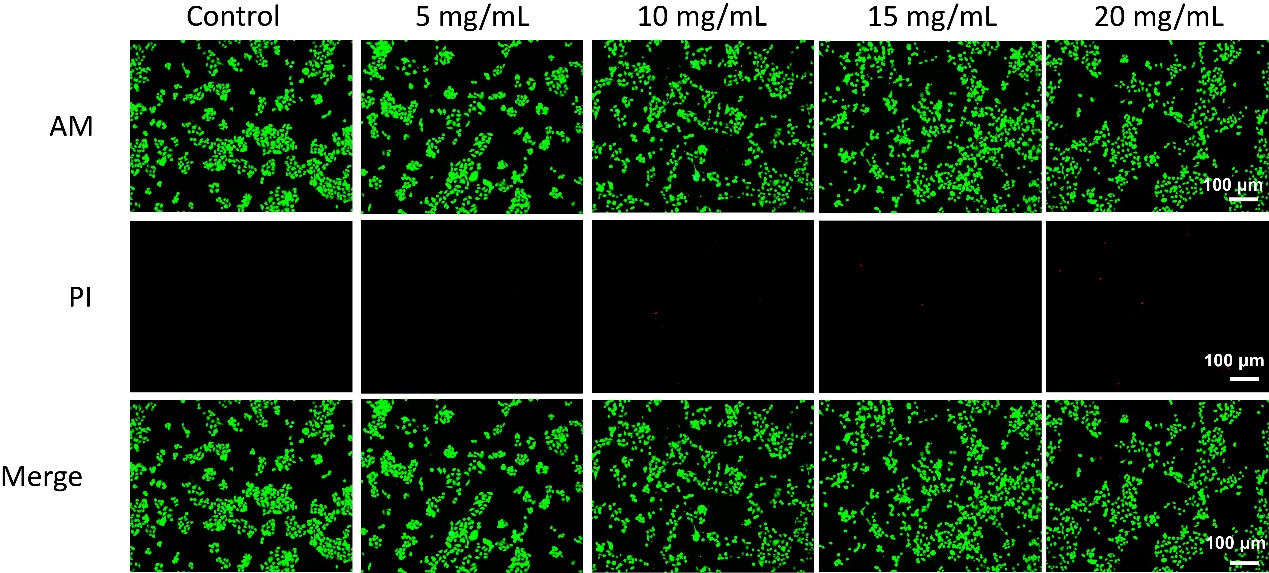


**Figure S13.** Live/Dead Cell Staining of HFE-145 cells after co-culturing with *L. reuteri*@HTP with different concentrations for 1 day.

**
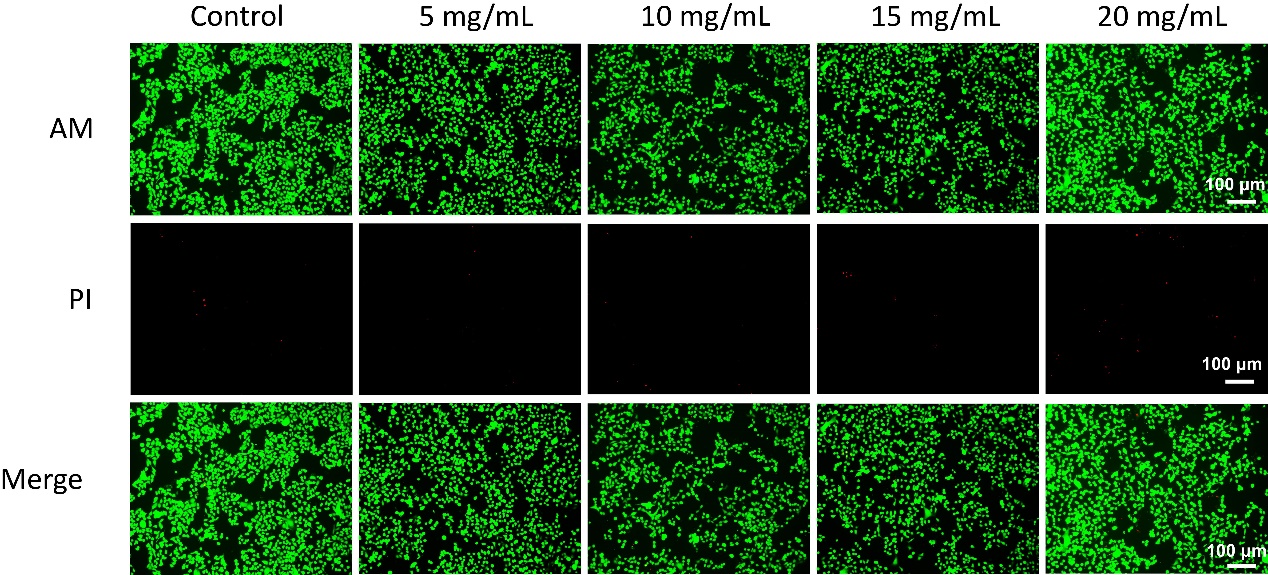
**

**Figure S14.** Live/Dead Cell Staining of HFE-145 cells after co-culturing with *L. reuteri*@HTP with different concentrations for 2 days.


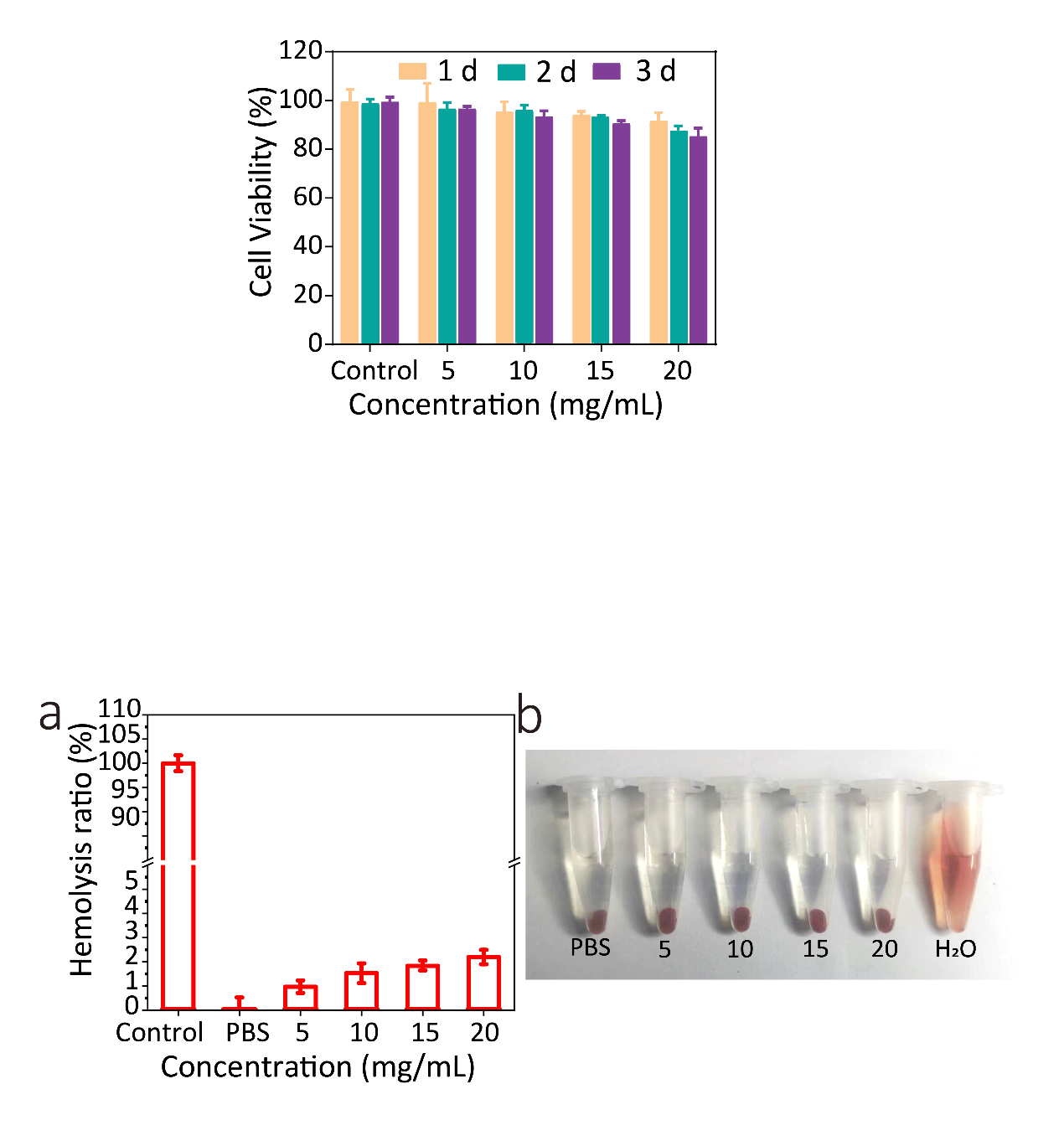


**Figure S15.** The viability of HFE-145 cells after coculture with *L. reuteri*@HTP at different concentrations for 1, 2, and 3 days. Data are presented as the mean ± SD (n = 3).


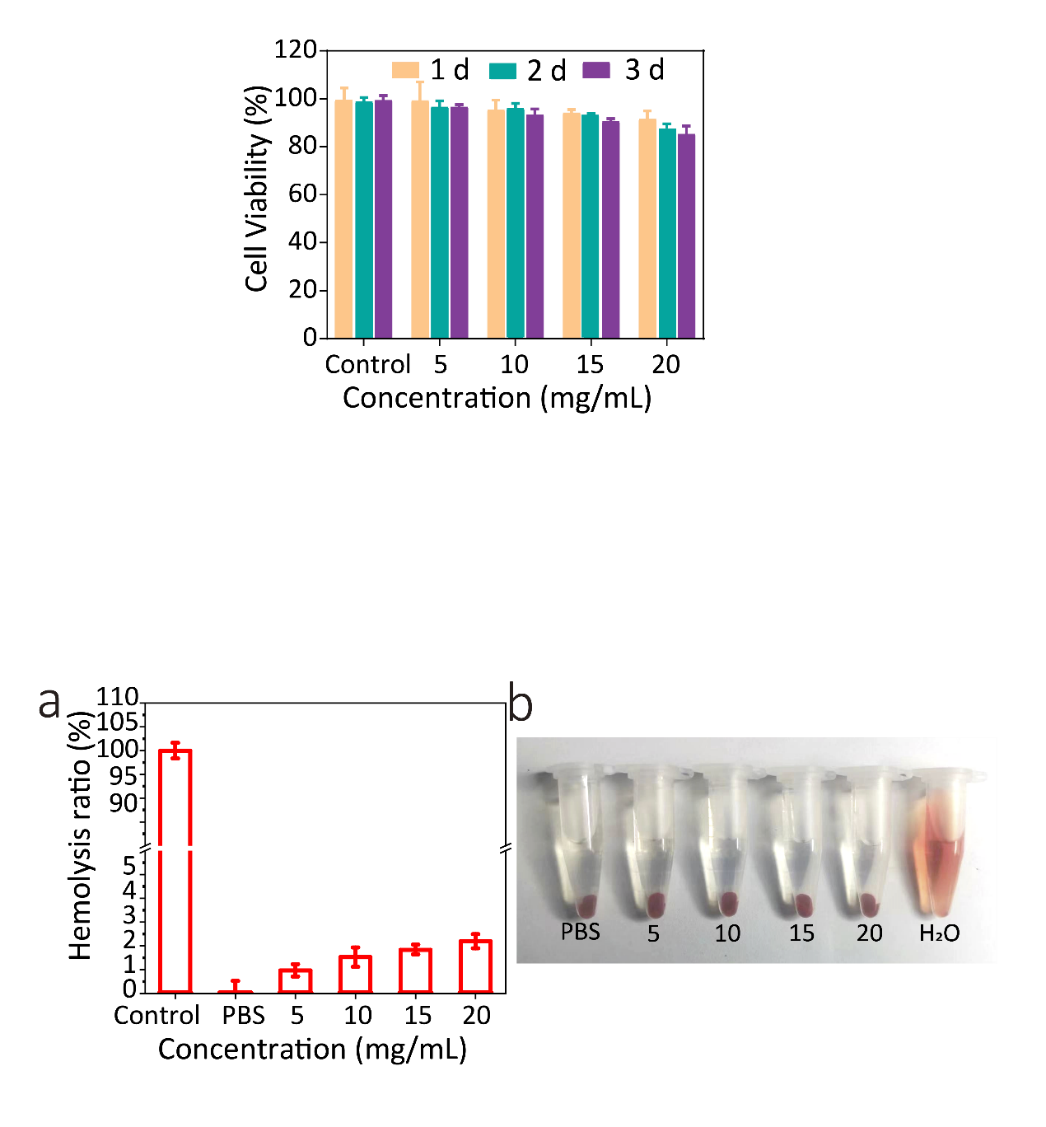


**Figure S16.** Hemolysis rates (a) and corresponding optical imaging (b) were evaluated for *L. reuteri*@HTP across a range of concentrations. Data are presented as the mean ± SD (n = 3).


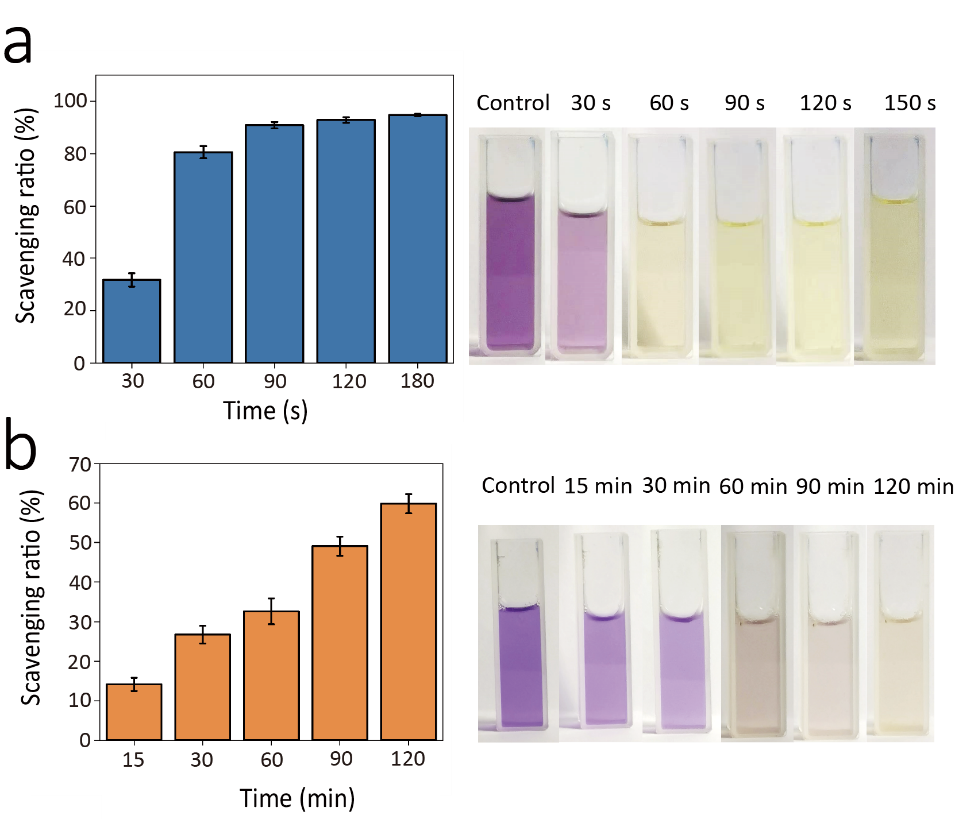


**Figure S17.** (a) DPPH· and (b) PITO· scavenging ratio and digital photographs of the scavenging process of HTP hydrogel. Data are presented as the mean ± SD (n = 3).


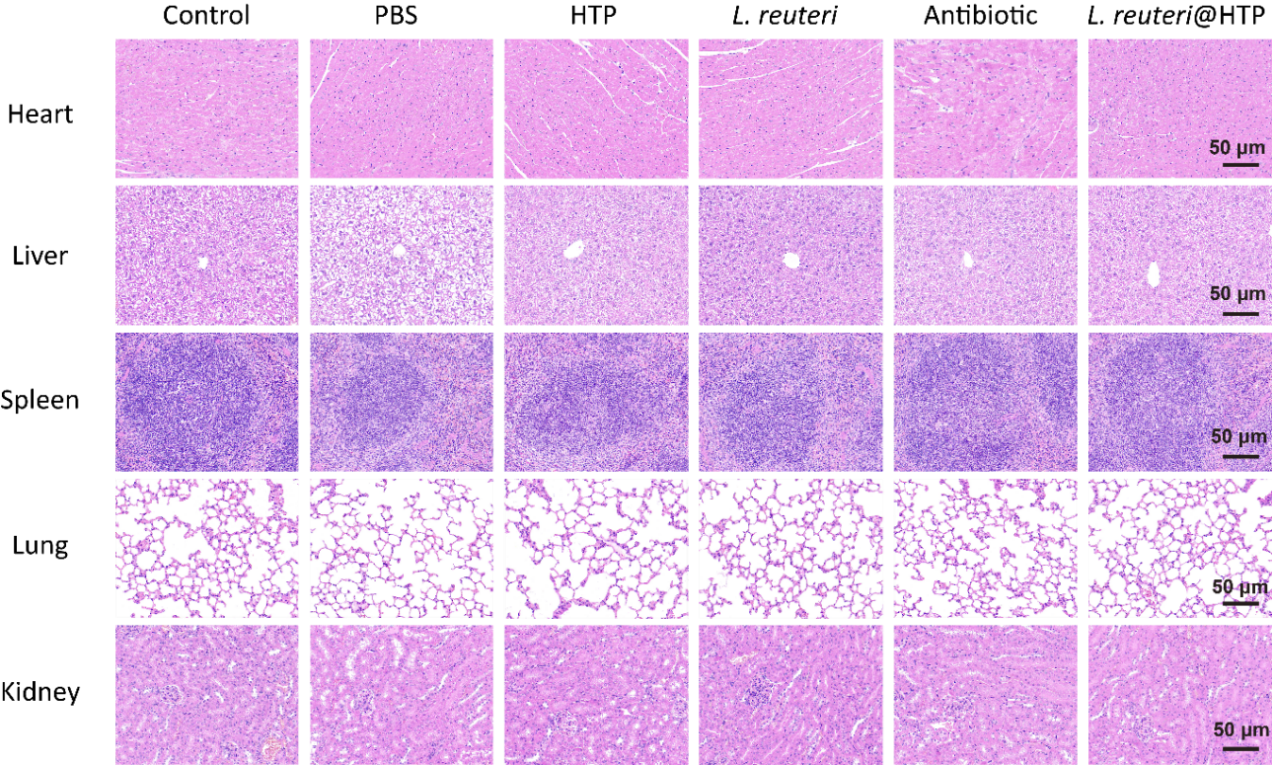


**Figure S18.** Hematoxylin and eosin (H&E) staining of the heart, liver, spleen, lung, and kidney of mice after treatment with PBS, HTP, *L. reuteri*, antibiotics, and *L. reuteri*@HTP powder for one week.


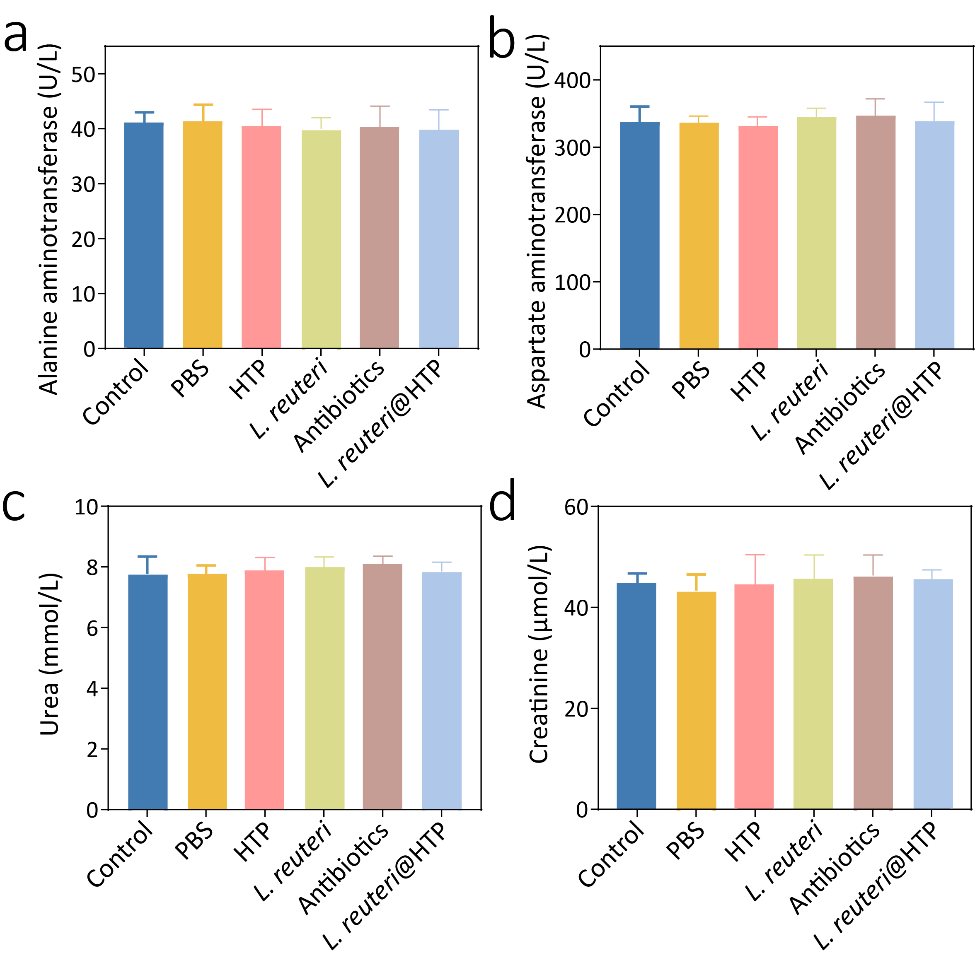


**Figure S19**. Liver and kidney function test results of mice after treatment with *L. reuteri*@HTP. Data are presented as the mean ± SD (n = 3).


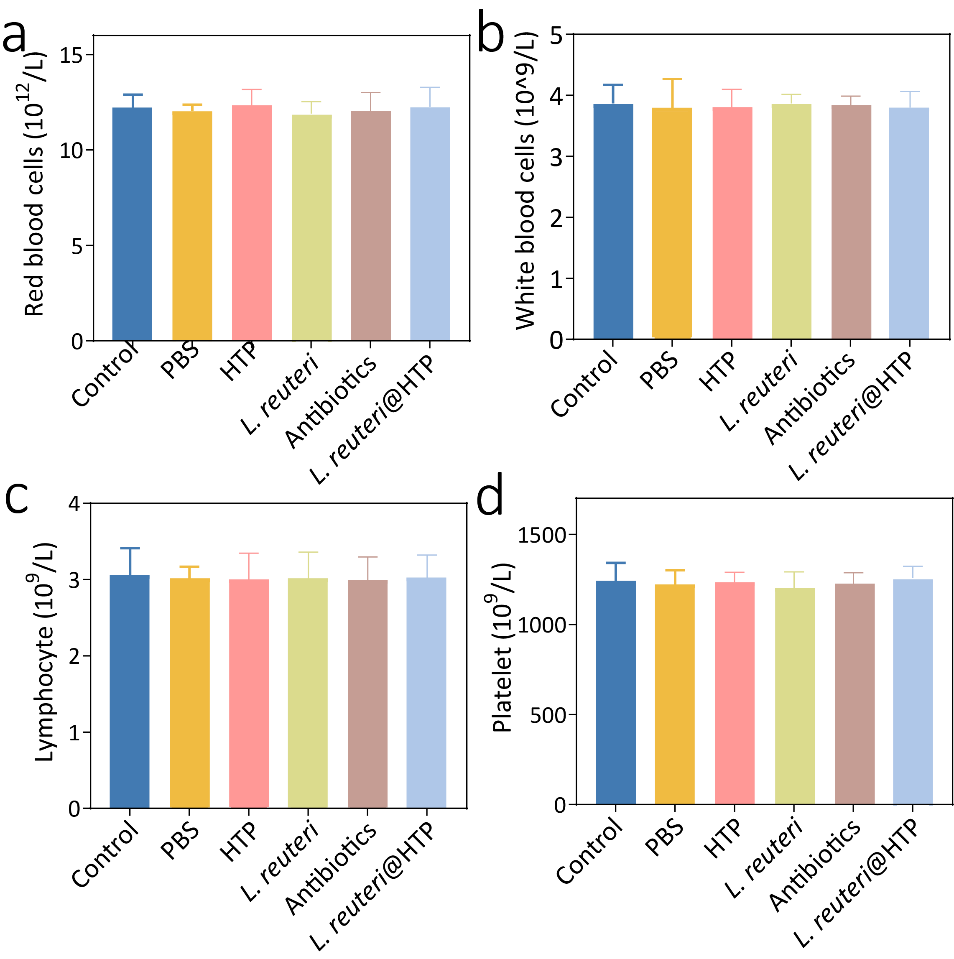


**Figure S20.** Routine blood test results of mice after *L. reuteri*@HTP treatment. Data are presented as the mean ± SD (n = 3).


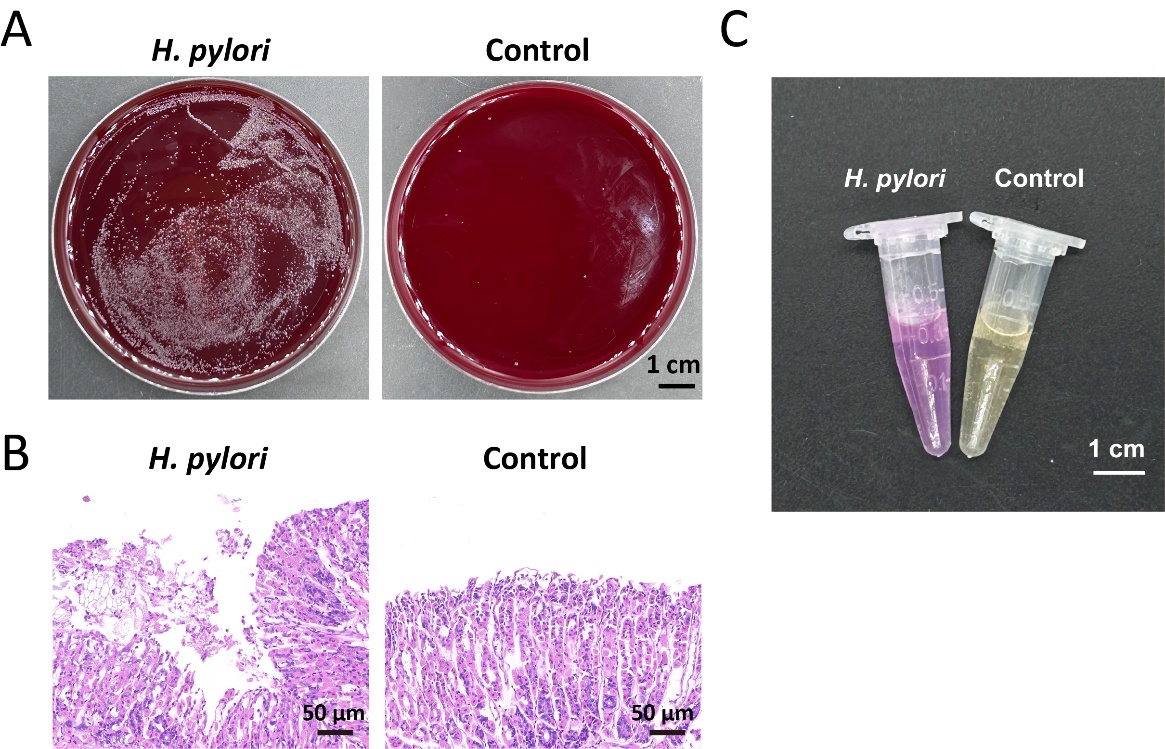


**Figure S21**. Validation of the *H. pylori*-infected mouse model. (A) Blood agar plate coating image, bars represent 1 cm. (B) H&E staining, bars represent 50 µm. (C) Urease test, bars represent 1 cm.


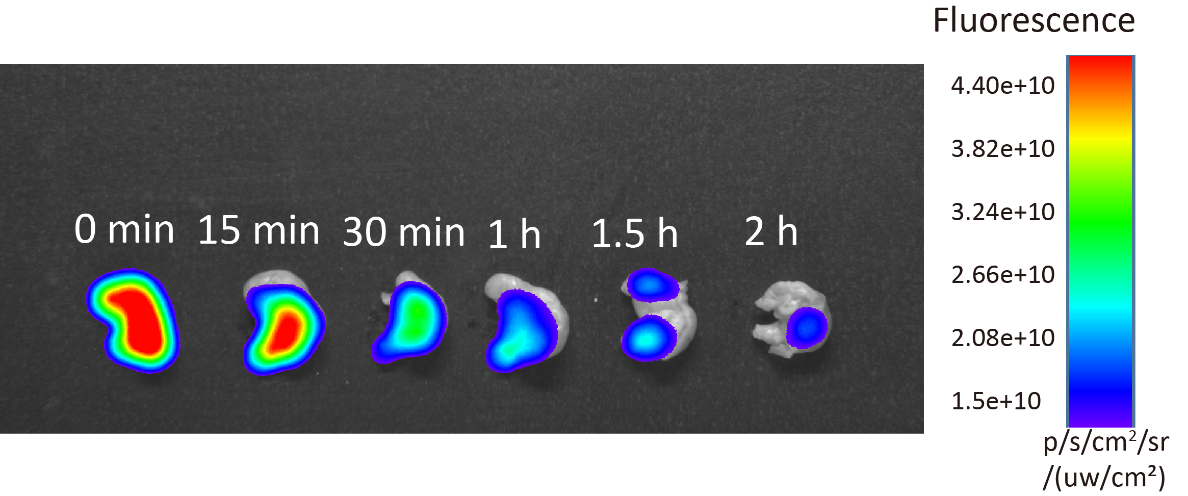


**Supplement Figure 22.** We labeled HTP with Cy7 and then encapsulated *L. reuteri* to prepare *L. reuteri*@HTP (Cy7-labeled). After a one-day fasting period, six *H. pylori*-infected mice were gavaged with *L. reuteri*@HTP(Cy7-labeled), and the mice were sacrificed at 0 min, 15 min, 30 min, 1 h, 1.5 h, and 2 h after gavage. Gastric tissues were imaged using the IVIS imaging system.


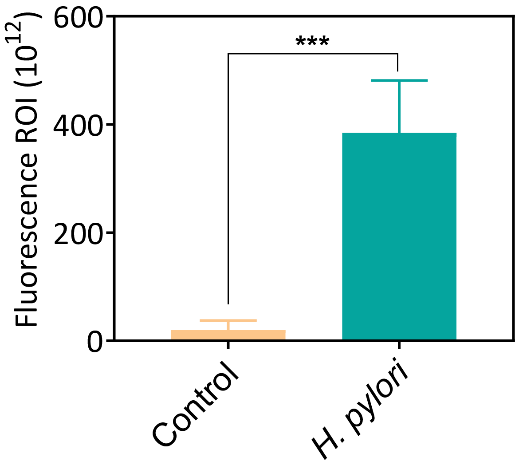


**Figure S23**. Quantitative analysis of the fluorescence intensity within the gastric tissues as visualized by the IVIS system-fluorescence images presented in Figure 6d. *H. pylori: H. pylori*-infected mice, Control: healthy mice, ROI: Region of Interest. Data are presented as the mean ± SD (n = 3), **p <* 0.05, ***p <* 0.01, and ****p <* 0.001.


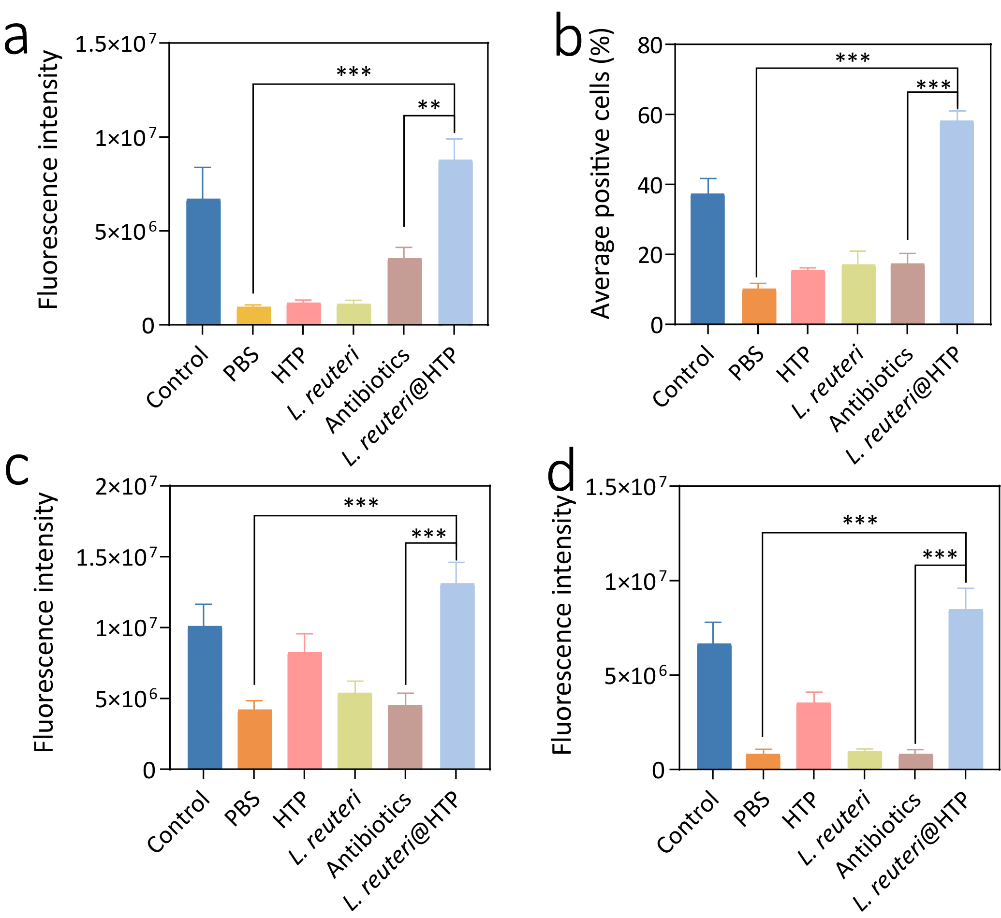


**Figure S24**. Quantitative immunofluorescence analysis results of (a)β-catenin, (b) PCNA, (c) Occludin, and (d) Claudin-1 of HEF-145 cells following various treatment modalities. Data are presented as the mean ± SD (n = 3), **p <* 0.05, ***p <* 0.01, and ****p <* 0.001.
